# FaNPR3 Members of the NPR1-like Gene Family Negatively Modulate Strawberry Fruit Resistance against *Colletotrichum acutatum*

**DOI:** 10.3390/plants13162261

**Published:** 2024-08-14

**Authors:** Victoria Súnico, José Javier Higuera, Francisco Amil-Ruiz, Isabel Arjona-Girona, Carlos J. López-Herrera, Juan Muñoz-Blanco, Ana María Maldonado-Alconada, José L. Caballero

**Affiliations:** 1 Biotechnology and Plant Pharmacognosy (BIO-278), Department of Biochemistry and Molecular Biology, Campus de Rabanales, Severo Ochoa building-C6, University of Córdoba, UCO-CeiA3, 14071 Córdoba, Spain; b12susam@uco.es (V.S.); b92hisoj@uco.es (J.J.H.); bb1mublj@uco.es (J.M.-B.); 2 Bioinformatics Unit, Central Research Support Service (SCAI), University of Córdoba, 14071 Córdoba, Spain; b72amruf@uco.es; 3 Department of Crop Protection, Institute for Sustainable Agriculture (CSIC), Alameda del Obispo s/n, 14004 Córdoba, Spain; isabelarjona@ias.csic.es (I.A.-G.); lherrera@ias.csic.es (C.J.L.-H.); 4 Agroforestry and Plant Biochemistry, Proteomics and Systems Biology, Department of Biochemistry and Molecular Biology, University of Cordoba, UCO-CeiA3, 14014 Córdoba, Spain

**Keywords:** *Fragaria ananassa*, strawberry defense, strawberry resistance, *Colletotrichum acutatum*, FaNPR3, *NPR*-like genes, AtNPR3, AtNPR4, FaWRKY19, FaWRKY24

## Abstract

Strawberry fruit is highly appreciated worldwide for its organoleptic and healthy properties. However, this plant is attacked by many pathogenic fungi, which significantly affect fruit production and quality at pre- and post-harvest stages, making chemical applications the most effective but undesirable strategy to control diseases that has been found so far. Alternatively, genetic manipulation, employing plant key genes involved in defense, such as members of the NPR-like gene family, has been successful in many crops to improve resistance. The identification and use of the endogenous counterpart genes in the plant of interest (as it is the case of strawberry) is desirable as it would increase the favorable outcome and requires prior knowledge of their defense-related function. Using RNAi technology in strawberry, transient silencing of *Fragaria ananassa* NPR3 members in fruit significantly reduced tissue damage after *Colletotrichum acutatum* infection, whereas the ectopic expression of either *FaNPR3.1* or *FaNPR3.2* did not have an apparent effect. Furthermore, the ectopic expression of *FaNPR3.2* in *Arabidopsis thaliana* double-mutant *npr3npr4* reverted the disease resistance phenotype to *Pseudomonas syringe* to wild-type levels. Therefore, the results revealed that members of the strawberry FaNPR3 clade negatively regulate the defense response to pathogens, as do their Arabidopsis AtNPR3/AtNPR4 orthologs. Also, evidence was found showing that FaNPR3 members act in strawberry (*F. ananassa*) as positive regulators of *WRKY* genes, *FaWRKY19* and *FaWRKY24*; additionally, in Arabidopsis, FaNPR3.2 negatively regulates its orthologous genes *AtNPR3/AtNPR4*. We report for the first time the functional characterization of FaNPR3 members in *F. ananassa*, which provides a relevant molecular basis for the improvement of resistance in this species through new breeding technologies.

## 1. Introduction

Strawberry (*Fragaria* spp.) represents a valuable and important food crop, with its fruit being highly appreciated by consumers worldwide [1]. Beyond its nutritional interest and sensory attributes, such as texture, color, flavor, and aroma, strawberry fruit provides substantial health benefits. This is due to the presence of bioactive compounds with a range of functions like control of blood glucose level, high antioxidant capacity, and potential cancer-prevention effects [2,3,4,5].

Strawberry is vulnerable to a wide diversity of pathogens, including fungi, which significantly affect fruit yield and quality, and *Colletotrichum acutatum*, is considered a serious fungal pathogen of this crop, causing severe losses in production [6,7,8,9,10]. The lack of pathogen-resistant cultivars is a major challenge for strawberry cultivation, making chemical applications the more widely used strategy to control diseases, endangering ecological and food security [11]. Indeed, classical breeding for resistance is an arduous task in strawberry due to the polygenic and quantitative inherited nature of this trait and due to the complexity of the octoploid genome of commercial varieties (*Fragaria* × *ananassa*) [12,13,14,15,16]. Therefore, innovative solutions for a more sustainable strawberry production are highly in demand.

Although many environmentally friendly strategies have been applied in strawberry to overcome susceptibility to pathogens [17,18,19,20,21,22], the genetic modification approaches using key genes that control the main defense pathways in plants has led to improving resistance in many crops [23,24,25,26,27]; and the heterologous expression of defense genes from other species, including those encoding chitinases, beta-1,3-glucanases and thaumatin II, has been reported in strawberry to successfully enhance resistance [28,29,30,31,32,33,34,35]. More recently, new breeding strategies have emerged as important tools to accelerate crop improvement based on powerful biotechnological techniques such as CRISPR/Cas and novel concepts of cisgenesis and intragenesis, assisted by synthetic biology [36,37,38,39,40], which have provided a valuable and more ecological framework for strawberry disease management than the chemical agents used until now [41]. However, it requires the prior identification and characterization of the appropriate endogenous candidate genes [14,42].

Over the last two decades, studies on genome structure and strawberry–pathogen interactions have provided new molecular information on the putative components of the main defensive pathways in this crop [43,44,45,46,47,48,49,50,51,52]. Nevertheless, accurate defense pathways remain yet elusive in strawberry and key defense network elements are still largely unknown and need to be functionally validated. For this reason, our research focuses on delving into endogenous strawberry genes homologous to crucial components of the defense pathways known in other plants, with the aim of characterizing and functionally validating valuable genetic elements to accelerate the improvement of resistance in this crop.

In this sense, members of the nonexpressor of pathogenesis-related gene family (NPR gene family) have been described as important molecular elements in the immune responses in plants. Indeed, *Arabidopsis thaliana* (Arabidopsis) AtNPR1 has been identified as a master regulator of salicylic acid (SA)-mediated defense responses [53,54]. AtNPR family differentiates into clade I (NPR1 and NPR2), clade II (NPR3 and NPR4), and clade III (NPR5/BOP2 and NPR6/BOP1) where only members of clade I and II have been associated with defense. Thus, single mutants *npr1* and *npr2* show increased susceptibility to *Pseudomonas syringe* (*Pst*) pathogen infection, whereas double mutant *npr3npr4* displays enhanced resistance [55]. AtNPR1 protein acts as a “sensor” of SA and, in response to SA-induced redox changes, is released from an inactive oligomeric cytoplasmic state and translocated as a monomeric form to the nucleus where, in association with transcription factors like TGAs, activate defense-related genes [53,56,57,58]. On the other hand, AtNPR3 and AtNPR4 also interact with TGAs; but, in contrast to AtNPR1, after binding SA, they negatively regulate the immune response. Thus, it has been proposed that AtNPR3/4 may act directly as transcriptional corepressors of defense-related genes [59] and also mediate the degradation of AtNPR1 and defense-related JAZs (Jasmonate-zim represor proteins), acting as adaptors for the ubiquitin ligase (CRL3) complex to enhance defense against pathogens [53,60,61].

The wide relevance of NPR family members in plant defense is evidenced by the fact that orthologs of all three AtNPR clades are conserved in most angiosperm species. Furthermore, the ectopic expression of AtNPR1 in many crop species, including strawberry, enhances broad-spectrum disease resistance, suggesting the existence of closely related defense response mechanisms among these species [62,63,64,65,66,67,68]. However, the molecular functions of AtNPR1 and its paralogs are still not fully understood and increased resistance using Arabidopsis AtNPR members often correlates with undesirable fitness costs [53,69,70,71], which highlights the importance of using functionally well-characterized endogenous NPR orthologs to engineer improved resistance in crops.

Recently, a comprehensive identification and phylogenic analysis of the strawberry NPR-like family has been published [49]. Accordingly, 6 NPR-like members are present in the diploid species (*Fragaria vesca*) and 23 members in cultivated species (*F. ananassa*), with each *F. vesca* member matching 3 to 5 homoalleles in the cultivated species and each clade headed by two Arabidopsis orthologs. Thus, the unique clade I member in *F. vesca*, FveNPR1, and its five homoalleles in *F. ananassa*, FaNPR1a-e, display high degree of identity among them and the Arabidopsis clade I members, AtNPR1 and AtNPR2. Similarly, the *F. vesca* clade II members, FveNPR31, FveNPR32, and FveNPR33, and their corresponding *F. ananassa* homoalleles, FaNPR31a-c, FaNPR32a-g, and FaNPR33a-d, share identity highly with Arabidopsis clade II members, AtNPR3 and AtNPR4. Finally, clade III FveNPR5 members in the diploid species and its *F. ananassa* four homoalleles, FaNPR5a-d, share identity highly with Arabidopsis clade III members, AtNPR5 and AtNPR6. Based on these comparative molecular studies and on the transcriptomic profile of these genes in response to *Colletotrichum fructicola*, the potential orthologues of AtNPR1 and AtNPR3/4 were identified in strawberry.

Despite these studies, the molecular functions of strawberry NPR-like members in promoting plant survival against pathogens remains uncovered. Just a first approach to functional characterization of the *F. vesca* gene *FvNPR31* by its ectopic expression in a heterologous system such as wild-type Arabidopsis has been published [72]. In the present work, we describe for the first time the functional characterization in the cultivated species *F. ananassa* of members of clade II NPR-like gene family using RNAi technology and gene complementation studies in Arabidopsis double mutant *npr3npr4*. Our results show that members of the FaNPR3 clade negatively regulate the defense response to pathogens, as do their Arabidopsis AtNPR3/AtNPR4 orthologs.

## 2. Results

### 2.1. Expression Pattern of FaNPR Gene Family after Transient Silencing and Overexpression of the FaNPR3 Members in Strawberry Fruit

To understand the biological role that the FaNPR3 family plays in strawberry defense, a transient expression-induced gene silencing and overexpressing approach in fruit was used. Thus, for silencing, one of the two halves of a fruit was infiltrated with *Agrobacterium tumefaciens* (agroinfiltrated), carrying the silencing construct (either pFRN::FaNPR3*all*.RNAi or pB7GWIWG2::FaNPR32.RNAi), and the opposite half of the same fruit was infiltrated with the control construct (either pFRN or pB7GWIWG2). Two days after agroinfiltration (dai), both halves of the fruit were inoculated with *C. acutatum*, (see experimental design in Material and Methods). Changes in the expression pattern of all *FaNPR* genes for every experimental condition were analyzed by qRT-PCR.

**Figure 1:**
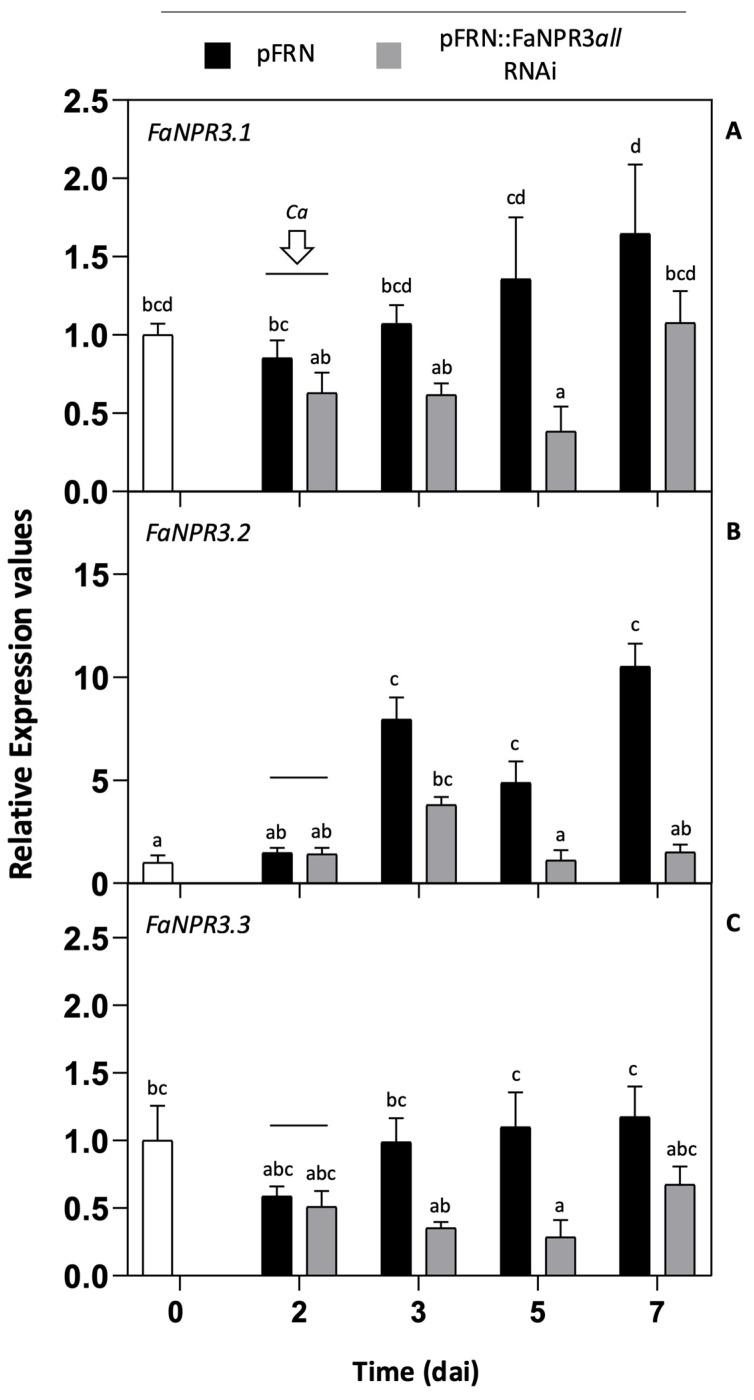
Expression pattern of *FaNPR3* genes in strawberry fruit after agroinfiltration with pFRN::FaNPR3*all*.RNAi construct. (**A**) Gene expression of *FaNPR3.1*. (**B**) Gene expression of *FaNPR3.2*. (**C**) Gene expression of *FaNPR3.3*. qRT-PCR analysis was accomplished in agroinfiltrated strawberry fruit before (0 and 2 days) and after (3, 5, and 7 days) *C. acutatum* inoculation (*Ca*). The time scale refers to days after agroinfiltration (dai). For all the genes, the arrow and horizontal line indicate the time of *Ca* inoculation. The black and grey columns show pFRN (control) and pFRN::FaNPR3*all*.RNAi (silencing) agroinfiltrations, respectively. Data from all time points are referred to data in time zero, represented as 1 (white column). Bars, mean ± standard error. Note the different scales in the relative-expression-level axis. Statistical significance was determined by one-way ANOVA. Letters indicate significant differences (*p*  <  0.05) in HSD Tukey’s post hoc test.

Two days after agroinfiltration and throughout the time analyzed, a significant decrease in *FaNPR3.1* transcript accumulation in pFRN::FaNPR3*all*.RNAi samples was detected compared to that of their corresponding pFRN control ones (Figure 1A). The expression of *FaNPR3.1* decreased to a lower significant level at 5 dai, which corresponds to 3 days post-inoculation with *C. acutatum* (3 dpi). The expression patterns of genes *FaNPR3.2* and *FaNPR3.3*, respectively, were similar to that of *FaNPR3.1*; a significant reduction in transcript accumulation was detected for both genes after 2 dai in the fruit half agroinfiltrated with the silencing construct compared with the control (Figure 1B,C). As for *FaNPR3.1*, a remarkable and significant decrease in transcript accumulation was observed around 5 dai in *FaNPR3.2* and *FaNPR3.3* genes. On the contrary, no significant differences in the expression level of other members of the *FaNPR* gene family, such as *FaNPR1* and *FaNPR5* genes, were detected between the control fruit and the silenced fruit samples. On the other hand, in the control fruit samples, a relevant induction in the expression of *FaNPR3.2* was detected after *C. acutatum* inoculation, which remained significantly high even after 5 dpi (Figure 1B), compared to that of genes *FaNPR3.1* and *FaNPR3.3* (Figure 1A,C), which indicates that *FaNPR3.2* gene members are preferentially activated in response to pathogen infection. Under these experimental conditions, similar patterns of gene silencing were detected for all the *FaNPR3* genes in fruit agroinfiltrated with the silencing construct, regardless of whether or not it had been inoculated with *C. acutatum*. All in all, these results revealed that, after fruit agroinfiltration, the pFRN::FaNPR3*all*.RNAi construct was successful in silencing, specifically the three *FaNPR3* variants of the *NPR*-like gene family.

**Figure 2:**
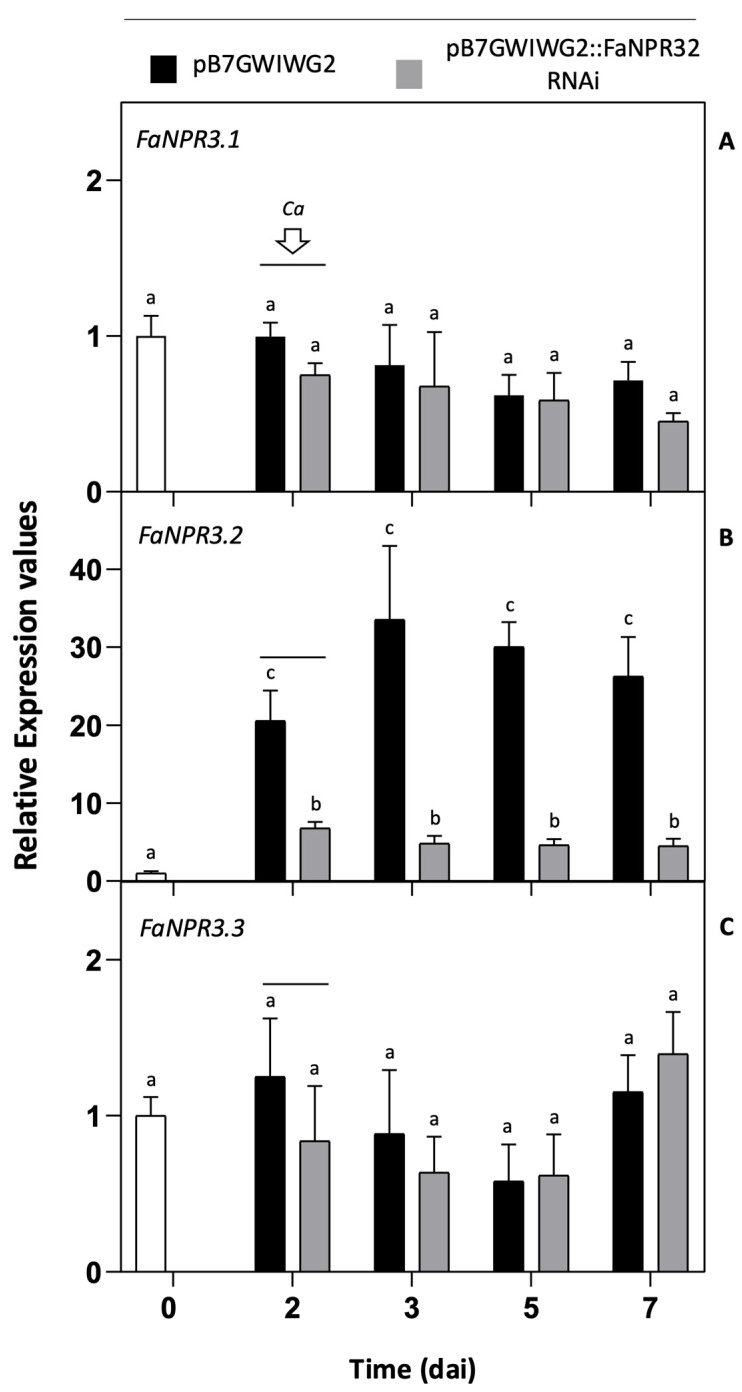
Expression pattern of *FaNPR3* genes in strawberry fruit after agroinfiltration with pB7GWIWG2::FaNPR32.RNAi. (**A**) Gene expression of *FaNPR3.1*. (**B**) Gene expression of *FaNPR3.2*. (**C**) Gene expression of *FaNPR3.3*. qRT-PCR analysis was accomplished in agroinfiltrated strawberry fruit before (0 and 2 days) and after (3, 5, and 7 days) *C. acutatum* inoculation (*Ca*). The time scale refers to days after agroinfiltration (dai). For all the genes, the arrow and the horizontal line indicate the time of *Ca* inoculation. The black and grey columns show pB7GWIWG2 and pB7GWIWG2::FaNPR32.RNAi agroinfiltrations, respectively. Data from all time points are referred to data in time zero, represented as 1 (white column). Bars, mean ± standard error. Note the different scales in the relative-expression-level axis. Statistical significance was determined by one-way ANOVA. Letters indicate significant differences (*p*  <  0.05) in HSD Tukey’s post hoc test.

Thus, a significant reduction in transcript accumulation of *FaNPR3.2* was detected after 2 dai in the fruit half sample agroinfiltrated with this construct as compared to its corresponding opposite fruit half agroinfiltrated with the control vector pB7GWIWG2 (Figure 2B). This significant reduction was evident for up to 7 dai (5 dpi). However, no significant changes in transcript accumulation were detected for *FaNPR3.1* (Figure 2A) and *FaNPR3.3* (Figure 2C) genes at any time sampled when comparing the fruit half silenced with its corresponding fruit half control. Similarly to the results shown in Figure 1B, after *C. acutatum* inoculation, a relevant induction in the expression of *FaNPR3.2* was detected in control fruit samples (Figure 2B), whereas no significant changes in gene expression were detected for *FaNPR3.1* and *FaNPR3.3* (Figure 2A and Figure 2C, respectively), which strongly support that *FaNPR3.2* is preferentially activated in response to pathogen infection. As aforementioned, under these experimental conditions, no relevant changes in transcript accumulation were found for any of the other gene members of the *NPR*-like family, *FaNPR1* and *FaNPR5*; and similar patterns of gene silencing were detected for all the *FaNPR3* genes when the agroinfiltrated fruit was not inoculated with *C. acutatum*. These results evidence that silencing of *FaNPR3.2* is mostly achieved in strawberry fruit after agroinfiltration with the pB7GWIWG2::FaNPR32.RNAi construct.

The effect of ectopic overexpression of *FaNPR3.1* and *FaNPR3.2* genes in strawberry fruit on the transcriptional pattern of all strawberry NPR-like gene family members was also analyzed using the pK7WG2::FaNPR31.OE and pB7WG2::FaNPR32.OE constructs for transient fruit expression, respectively, which carry the inserted genes under the control of the strong promoter CaMV35S. In our experimental conditions, the transient overexpression of either *FaNPR3.1* or *FaNPR3.2* variants (see Supplementary Materials Figure S1) in strawberry fruit did not significantly alter the transcript accumulation level of any other endogenous member of the *FaNPR* gene family.

### 2.2. The Silencing of FaNPR3 Genes in Strawberry Fruit Reduced Fruit Tissue Damage after C. acutatum Inoculation

**Figure 3:**
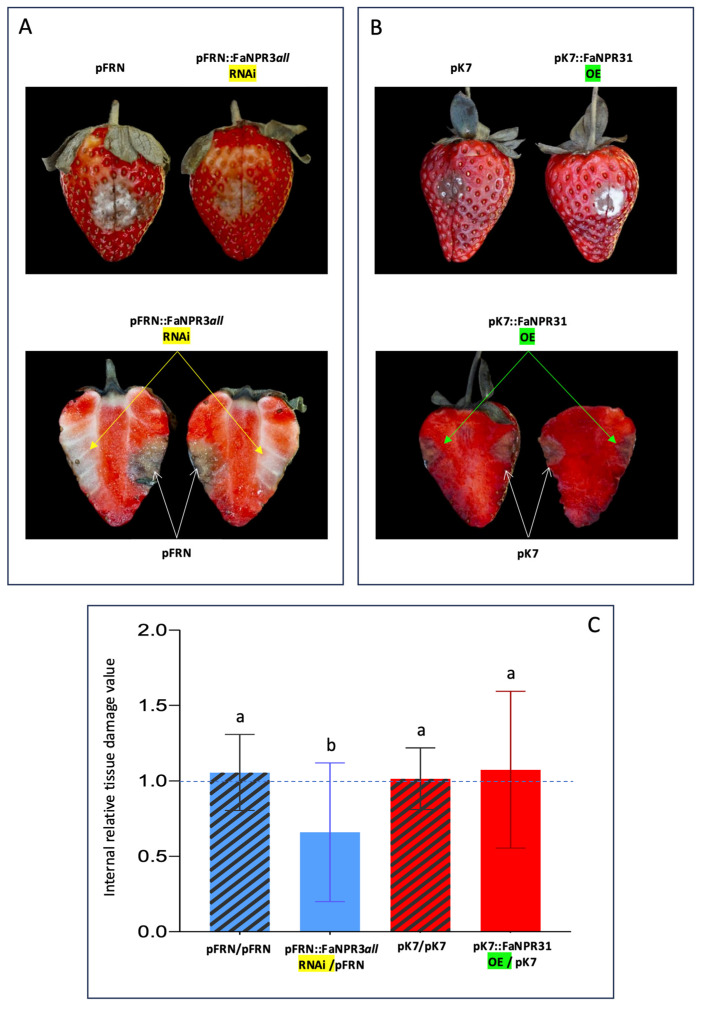
Silencing effect of pFRN::FaNPR3*all*.RNAi and ectopic overexpression of *FaNPR3.1* in strawberry fruit after *C. acutatum* infection. (**A**,**B**) Upper panels: external surface disease symptoms on the two agroinfiltrated opposite halves of the same fruit, after silencing and overexpression, respectively. (**A**,**B**) Lower panels: internal tissue damage of the same fruit shown in the corresponding upper panels. pFRN::FaNPR3*all*.RNAi and pFRN, silencing construct and its corresponding empty vector, as control. pK7::FaNPR31.OE and pK7, overexpression construct and its corresponding empty vector, as control. A relevant fruit is shown for each condition, as an example. (**C**) Statistical analysis of internal tissue damage ratio of the two opposite halves of the same fruit, according to the 1 to 5 severity scale; striped and plain blue bars, pFRN/pFRN and pFRN/pFRN::FaNPR31-RNAi agroinfiltrated values, respectively; striped and plain red bars, pK7/pK7 and pK7/pK7::FaNPR31-OE agroinfiltrated values, respectively. Data correspond to mean ± SD. Within each bar, means with different letters are significantly different by LSD test at *p* < 0.05. A ratio value of 1 indicates no differences between the opposite halves of the same fruit.

**Figure 4:**
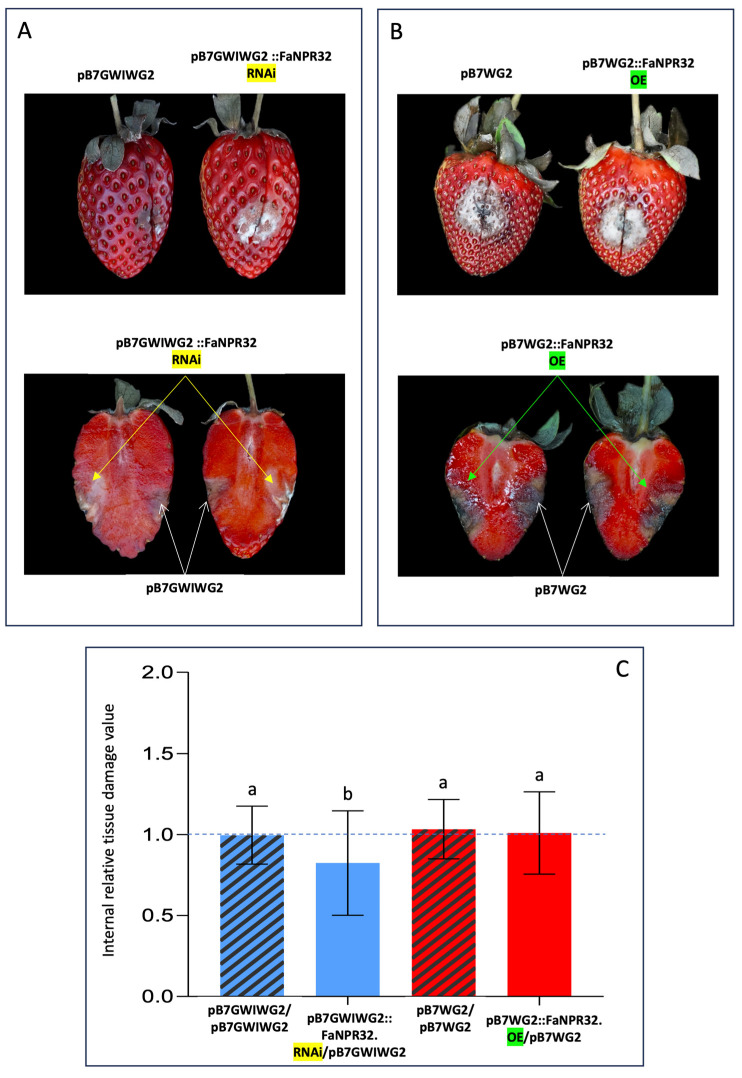
Silencing effect of pB7GWIWG2::FaNPR32.RNAi and ectopic overexpression of *FaNPR3.2* in strawberry fruit after *C. acutatum* infection. (**A**,**B**) Upper panels: external surface disease symptoms on the two agroinfiltrated opposite halves of the same fruit, after silencing and overexpression, respectively. (**A**,**B**) Lower panels: internal tissue damage of the same fruit shown in the corresponding upper panels. pB7GWIWG2::FaNPR32.RNAi and pB7GWIWG2, silencing construct and its corresponding empty vector, as control. pB7WG2::FaNPR32.OE and pB7WG2, overexpression construct and its corresponding empty vector, as control. A relevant fruit is shown for each condition, as an example. (**C**) Statistical analysis of internal tissue damage ratio of the two opposite halves of the same fruit, according to the 1 to 5 severity scale; striped and plain blue bars, pB7GWIWG2/pB7GWIWG2 and pB7GWIWG2/pB7GWIWG2::FaNPR32-RNAi agroinfiltrated values, respectively; striped and plain red bars, pB7WG2/pB7WG2 and pB7WG2/pB7WG2::FaNPR32-OE agroinfiltrated values, respectively. Data correspond to mean ± SD. Within each bar, means with different letters are significantly different by LSD test at *p* < 0.05. A ratio value of 1 indicates no differences between opposite halves of the same fruit.

According to the above results, the evaluation of fruit tissue damage and the comparative study of susceptibility to *C. acutatum* between the two opposite halves of the same fruit (one agroinfiltrated with either the silencing or the overexpression construct and the other with the corresponding empty vector as a control) were accomplished 6 dai. Overall, no relevant visual differences were observed in external tissue damage of opposite halves of the same fruit, in fruit samples silenced with the pFRN::FaNPR3*all*.RNAi or pB7GWIWG2::FaNPR32.RNAi silencing construct (upper panels, Figure 3A and Figure 4A, respectively), and fruit samples where *FaNPR3.1* or *FaNPR3.2* was overexpressed (upper panels, Figure 3B and Figure 4B, respectively). However, a relevant reduction in internal tissue damage was clear within a fruit half agroinfiltrated with the silencing construct versus a fruit half agroinfiltrated with control vector in both pFRN::FaNPR3*all*.RNAi (Figure 3A, lower panel) and pB7GWIWG2::FaNPR32.RNAi (Figure 4A, lower panel) silenced fruit samples. When genes *FaNPR3.1* and *FaNPR3.2* were ectopically overexpressed in strawberry fruit (lower panels of Figure 3B and Figure 4B, respectively), no relevant visual difference in internal tissue damage was observed within the fruit half agroinfiltrated with the overexpression construct compared to opposite half agroinfiltrated with the control vector.

A statistical analysis of the internal fruit tissue damage was conducted either in silenced or overexpressed fruit halves and compared to their corresponding control halves. The tissue damage value obtained by normalizing fruit halves transformed with the silencing construct, with respect to the corresponding opposite fruit halves transformed with the empty vector, were significantly reduced in the pFRN::FaNPR3*all*.RNAi- and pB7GWIWG2::FaNPR32.RNAi-silenced samples (mean values of 0.6603 and 0.8242, respectively) compared to those obtained when both fruit halves were agroinfiltrated with control constructs (mean values of 1.0556 and 0.9958, respectively) (Figure 3C and Figure 4C, left). However, no significant differences were found in *FaNPR3.1* and *FaNPR3.2* overexpressed samples (mean values of 1.0737 and 1.0097, respectively) compared to their corresponding control samples (mean values of 1.0152 and 1.0327, respectively) (Figure 3C and Figure 4C, right). For external fruit tissue damage, all the ratio values show no significant differences either for the silenced or the overexpressed samples. These results unravel a positive correlation between the silencing of members of the *FaNPR3* gene family and an increase in fruit resistance to *C. acutatum* infection.

### 2.3. Analysis of Defense-Related Genes in Strawberry Fruit Silenced in FaNPR3 Genes

**Figure 5:**
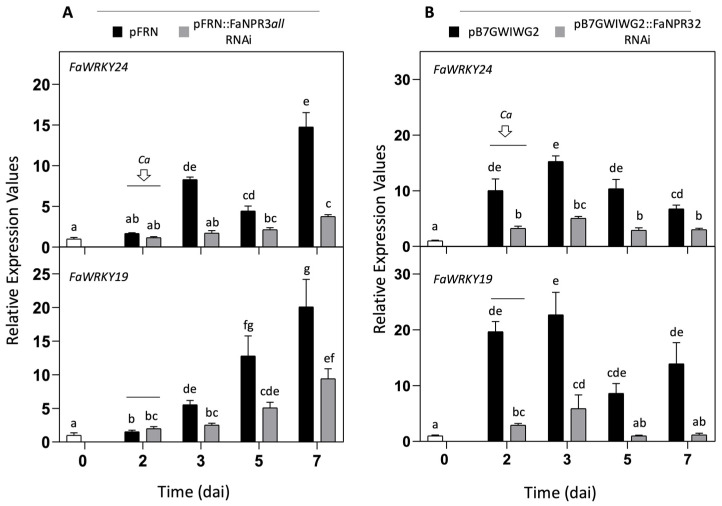
Silencing effect of *FaNPR3* members on the expression of *FaWRKY19* and *FaWRKY24* genes in strawberry fruit after *C. acutatum* infection. (**A**) Silencing effect of pFRN::FaNPR3*all*.RNAi. (**B**) Silencing effect pB7GWIWG2::FaNPR32.RNAi. Black columns, the expression of *FaWRKY19* and *FaWRKY24* genes in half fruit agroinfiltrated with pFRN (**A**) or pB7GWIWG2 (**B**) control vectors. Grey columns, the expression of *FaWRKY19* and *FaWRKY24* genes in half fruit agroinfiltrated with pFRN::FaNPR3*all*.RNAi (**A**) or pB7GWIWG2::FaNPR32.RNAi (**B**). qRT-PCR analysis was accomplished in agroinfiltrated strawberry fruit before (0 and 2 days) and after (3, 5, and 7 days) *C. acutatum* inoculation (*Ca*). The time scale refers to days after agroinfiltration (dai). Arrow and horizontal line indicate the time of *Ca* inoculation. Data from all time points are referred to data in time zero, represented as 1 (white column). Bars, mean ± standard error. Note the different scales in the relative-expression-level axis. Statistical significance was determined by one-way ANOVA. Letters indicate significant differences (*p*  <  0.05) in HSD Tukey’s post hoc test.

To gain insights into the strawberry defense network associated with members of the FaNPR3 clade, we analyzed the expression profile of several strawberry genes already known to respond to *C. acutatum* infection, such as *FaPR1*-1(*AtPR1* ortholog), *FaPR2*-1 (*AtPR2* ortholog), *FaPR5.2* (AT4G11650 ortholog), *FaWRKY19* (previously reported as *FaWRKY33-2*; *AtWRKY25/33/26* ortholog), *FaWRKY24* (previously reported as *FaWRKY1*; *AtWRKY75* ortholog), *FaWRKY41* (previously reported as *FaWRKY70-1*; *AtWRKY54/70* ortholog), and *FaWRKY60* (previously reported as *FaWRKY70-2*; *AtWRKY54/70* ortholog). The expression level of *FaWRKY19* and *FaWRKY24* genes increased in control fruit sample two days after agroinfiltration with either pFRN or pB7GWIWG2) and remained significantly high upon *C. acutatum* inoculation (Figure 5A,B). However, a significant reduction in the transcript accumulation was detected for both genes *FaWRKY19* and *FaWRKY24* in the corresponding opposite fruit half agroinfiltrated with the silencing constructs pFRN::FaNPR3*all*.RNAi (Figure 5A) and pB7GWIWG2::FaNPR32.RNAi (Figure 5B). Similar patterns of down-regulation were detected for *FaWRKY19* and *FaWRKY24* genes when the agroinfiltrated fruit was not inoculated with *C. acutatum*. For *FaPR1-1*, *FaPR2-1*, *FaPR5-2*, *FaWRKY41*, and *FaWRKY60* genes, no significant difference was found in their transcript accumulation patterns when comparing fruit agroinfiltrated with the silencing construct (either pFRN::FaNPR3*all*.RNAi or pB7GWIWG2::FaNPR32.RNAi) vs. its corresponding control construct.

These results provide strong evidence that the expression of genes *FaWRKY19* and *FaWRKY24* is positively modulated in fruit by FaNPR3 members of the strawberry NPR3-like gene family.

### 2.4. The Strawberry FaNPR3.2 Gene Complements the Arabidopsis Atnpr3npr4 Double Mutant Disease Resistance Phenotype

To obtain a deeper insight into the function of the strawberry FaNPR3 family, in parallel to the silencing of the *FaNPR3.1* and *FaNPR3.2* in strawberry, a heterologous complementation analysis has been conducted in Arabidopsis plants with *FaNPR3.2*.

Arabidopsis transgenic lines were generated by transforming WT and *npr3npr4* double mutant plants with pB7WG2::FaNPR32-OE, the DNA cassette driving the expression of the strawberry *FaNPR3.2* under the control of a strong promoter. Homozygous plants from each line were selected to perform functional complementation tests by characterizing the expression of the transgene and their disease resistance phenotype upon inoculation with virulent *Pseudomonas syringae* pathovar tomato strain DC3000 (*Pst*).

**Figure 6:**
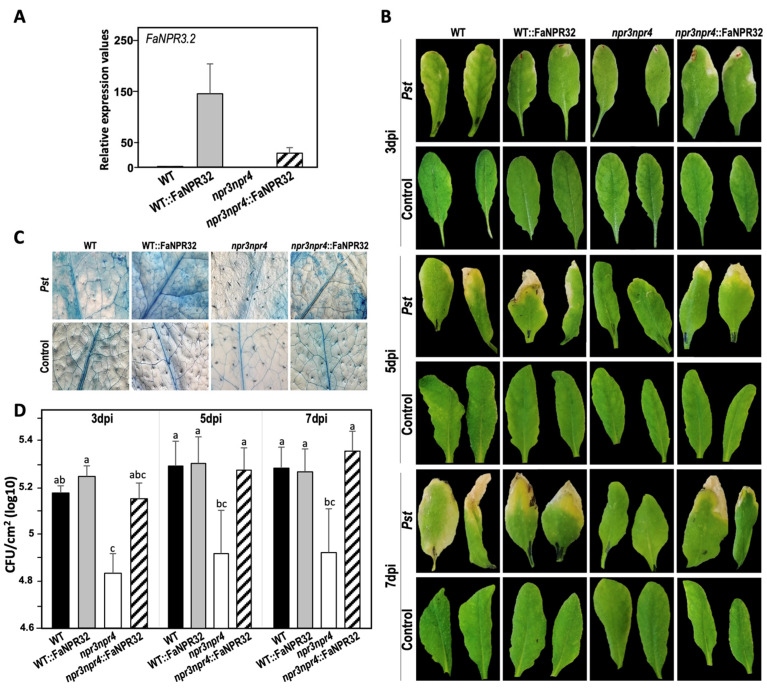
Characterization of Arabidopsis lines. (**A**) *FaNPR3.2* expression in Arabidopsis WT-, *npr3npr4* mutant-, and *FaNPR3.2*-overexpressing lines *npr3npr4*::FaNPR32 and WT::FaNPR32 (black, grey, white and striped bars, respectively). Transcript accumulation was monitored by qRT-PCR in non-infected plants as described in the Section 4. Expression levels were normalized with respect to the internal control *ACTIN2* and displayed relatively to the threshold value of the wild-type (no expression of *FaNPR3.2*) that was given a value of 1 for convenience. Note that no amplification of *FaNPR3.2* strawberry gene is detected in neither WT nor double mutant *npr3npr4* using the specific primers (Supplementary Materials Table S1). Bars represent the mean levels of transcript quantified from three independent biological experiments (±SD). (**B**–**D**) Disease resistance phenotype of Arabidopsis lines upon *Pst* inoculation. (**B**) Symptoms development on leaves 3, 5, and 7 days post-inoculation (dpi) (10^6^ CFU mL^−1^); (**C**) trypan blue staining for the detection of cell death 1 dpi (10^5^ CFU mL^−1^); (**D**) in planta bacterial growth monitored 3, 5, and 7 days post-inoculation (10^6^ CFU mL^−1^). CFU, colony-forming units. Statistically significant differences are labelled by letters (one-way ANOVA, Tukey’s multiple comparisons test, *p* < 0.05). *Pst* was pressure infiltrated into fully expanded mature leaves of 4–5-week-old Arabidopsis plants. As control, leaves were infiltrated with 10 mM MgCl_2_. The whole experiment was performed three times with similar results.

The *FaNPR3.2* transcript accumulated abundantly in the leaves of the overexpressing lines *npr3npr4*::FaNPR3.2 and even at higher levels in WT::FaNPR3.2 (Figure 6A). Interestingly, as shown in Figure 6B, the disease symptoms’ development at 3, 5, and 7 dpi on the leaves of *npr3npr4*::FaNPR3.2- and WT::FaNPR3.2-overexpressing transgenic plants was similar to that of WT plants, showing chlorotic symptoms and necrotic lesions, in contrast to the asymptomatic *npr3npr4* double mutant plants.

Next, the *Pst*-induced cell death phenotype by trypan blue staining was analyzed and plants overexpressing *FaNPR3.2* in the WT and *npr3npr4* mutant genetic background showed noticeably enhanced cell death compared to WT and double mutant *npr3npr4*, respectively (Figure 6C). Indeed, the presence of endogenous AtNPR3 and AtNPR4 in Arabidopsis together with the strawberry FaNPR3.2 resulted in cell death spreading across the entire infected leaves of WT::FaNPR3.2 plants, whereas it was more restricted in *npr3npr4*::FaNPR3.2 leaves. In addition, bacterial growth of virulent *Pst* was significantly restrained in the absence of AtNPR3 and AtNPR4 (double mutant *npr3npr4*) at all times tested (3, 5, and 7 dpi), while overexpression of *FaNPR3.2* reverted the WT disease phenotype in *npr3npr4* plants, displaying similar bacterial density than WT plants (Figure 6D).

Thus, Arabidopsis *npr3npr4*::FaNPR3.2 plants revert the *Pst*-increased resistance phenotype of the double mutant *npr3npr4* to wild-type levels, in terms of disease symptoms development and bacterial growth.

### 2.5. Changes in the Expression Profile of Defense-Related Genes in Arabidopsis

**Figure 7:**
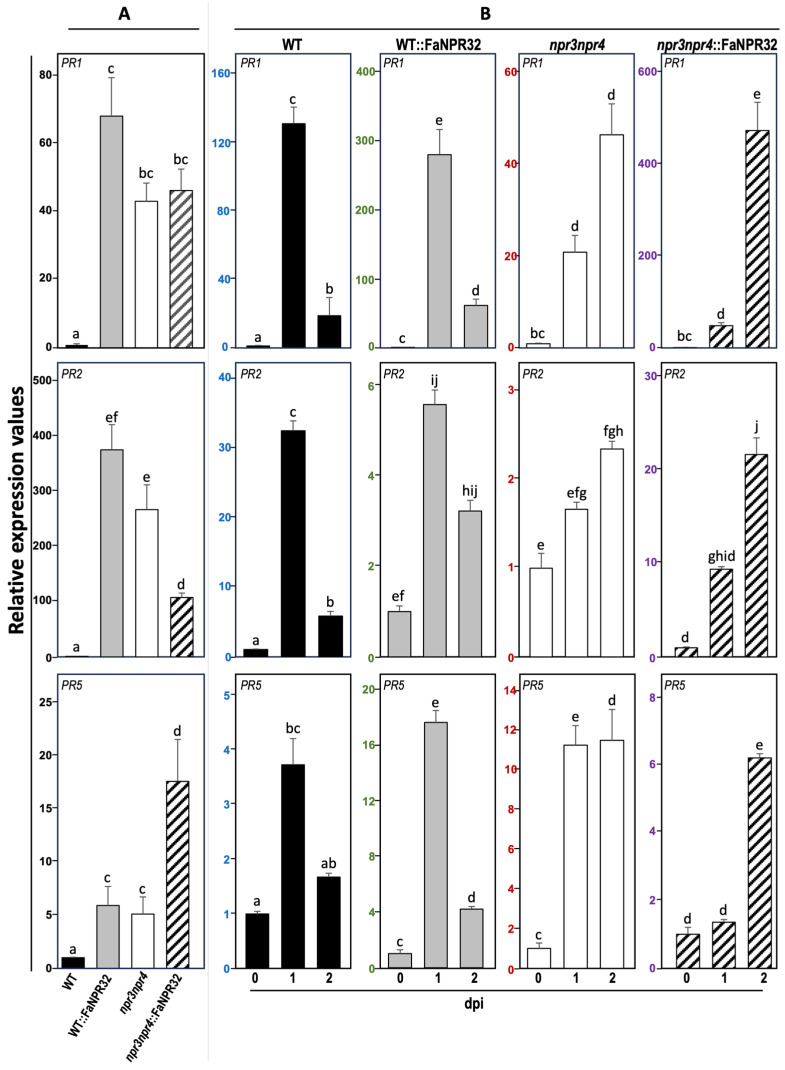
Expression of the defense-related genes *PR1*, *PR2*, and *PR5* in Arabidopsis lines. Relative expression level was monitored by qRT-PCR in control (**A**) and infected plants 1 and 2 days post-inoculation (dpi) with *Pst* (10^6^ CFU mL^−1^) (**B**), in WT, WT::FaNPR32, double mutant *npr3npr4*, and *npr3npr4*::FaNPR32 (black, grey, white and striped bars, respectively). Expression levels were normalized with respect to the internal control *ACTIN2* and displayed relative to the expression in mock-treated wild-type samples (**A**) or to the expression in mock-treated samples of each line (**B**) that were given a value of 1. Bars refer to mean ± standard error. Note the different scales in the relative-expression-level axis. Statistical significance was determined by one-way ANOVA. Letters indicate significant differences (*p*  <  0.05) in HSD Tukey’s post hoc test.

To shed light on the molecular mechanisms underlying FaNPR3.2 complementation of Arabidopsis double mutant *npr3npr4*, the expression profiles of the typical SA pathway defense marker genes *PR1*, *PR2*, and *PR5* were analyzed both at basal levels and after infection with *Pst* and compared among all the Arabidopsis lines. The results show that transcript accumulation of this set of genes was barely detectable in non-infected WT plants (Figure 7A), and it increased following infection (Figure 7B), indicating the activation of the defense response. However, in enhanced resistant *npr3npr4* mutant plants, the basal transcript level for these genes far exceeded that of WT in uninfected conditions, and was further strongly induced after *Pst* infection. Curiously, Arabidopsis plants overexpressing *FaNPR3.2*, which reverts the WT disease phenotype, displayed remarkably high basal levels of *PR*s transcript and these marker genes were even further induced upon *Pst* challenge (Figure 7B).

Our results reflect a clear uncoupling between the resistance phenotype and the induction of these defense-related markers in Arabidopsis plants overexpressing the strawberry *FaNPR3.2* gene.

**Figure 8:**
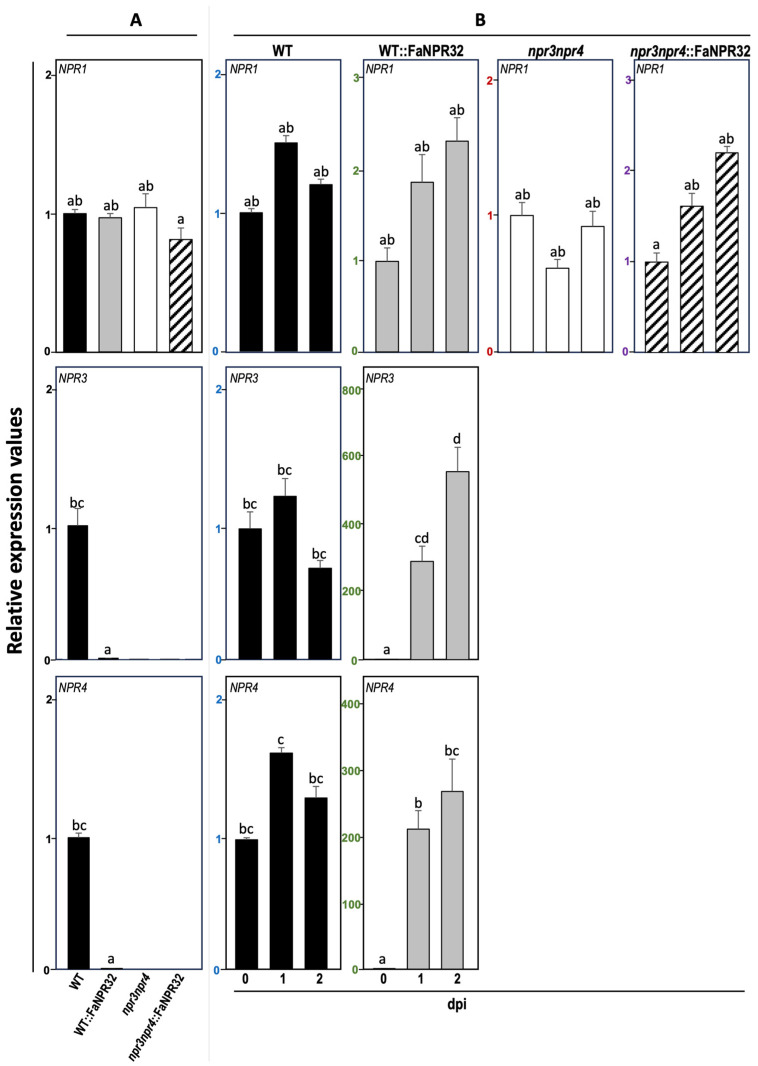
Expression of the endogenous *AtNPR1*, *AtNPR3*, and *AtNPR4* genes in Arabidopsis. Relative expression level was monitored by qRT-PCR in control (**A**) and infected plants 1 and 2 days post-inoculation (dpi) with *Pst* (10^6^ CFU mL^−1^) (**B**), in WT, WT::FaNPR32, double mutant *npr3npr4*, and *npr3npr4*::FaNPR32 (black, grey, white and striped bars, respectively). Expression levels were normalized with respect to the internal control *ACTIN2* and displayed relative to the expression in mock-treated wild-type samples (**A**) or to the expression in mock-treated samples of each line (**B**) that were given a value of 1. Bars refer to mean ± standard error. Note the different scales in the relative-expression-level axis. Statistical significance was determined by one-way ANOVA. Letters indicate significant differences (*p*  <  0.05) in HSD Tukey’s post hoc test. Note that the absence of transcript for *AtNPR3* and *AtNPR4* in the overexpressing lines in the double mutant *npr3npr4* background. It proves that Arabidopsis primers do not amplify strawberry orthologous genes.

Next, the question was explored of whether the enhanced basal level of these classic defense markers observed in lines overexpressing *FaNPR3.2* was related with an impaired expression profile of any of the endogenous members of the *NPR* gene family in Arabidopsis, *AtNPR1*, *AtNPR3*, and *AtNPR4*. Thus, the *NPR1* basal transcript level in uninfected plants was similar in WT, *npr3npr4* mutant, and their corresponding overexpressing FaNPR3.2 lines (Figure 8A), and also upon *Pst* infection, the *AtNPR1* gene expression remained unaltered in all lines tested (Figure 8B). As expected, no *AtNPR3* or *AtNPR4* transcripts were detected in *npr3npr4* or *npr3npr4*::FaNPR3.2 lines. Interestingly, the basal transcript level of both endogenous *AtNPR3* and *AtNPR4* was significantly reduced in WT::FaNPR3.2 compared to that of WT plants. (Figure 8A). In addition, a significantly stronger induction of *AtNPR3* and *AtNPR4* genes was observed in WT::FaNPR3.2-overexpressing lines compared to that of WT lines (Figure 8B).

The results shown in Figure 7A and Figure 8A evidence that, in the FaNPR3.2-overexpressing lines, a positive correlation exists between the enhanced basal level of *PR*s and the reduced basal level of *AtNPR3* and *AtNPR4*.

## 3. Discussion

### 3.1. Members of the FaNPR3 Clade Negatively Modulate Strawberry Fruit Resistance against Colletotrichum acutatum

This report primarily focuses on understanding the molecular role that *FaNPR3* members of the strawberry *NPR*-like gene family play in the defense response against pathogens. Thus, we have been quite successful in transiently silencing all members of *FaNPR3* clade II in strawberry fruit using *A. tumefaciens* carrying the silencing construct pFRN::FaNPR3*all*-RNAi (Figure 1). This construct contains a DNA sequence that was previously predicted to promote highly effective siRNAs matching all *FaNPR3* genes in strawberry (*F. ananassa*) [40]. Furthermore, we have also transiently silenced predominantly *FaNPR3.2* homeologs in strawberry fruit (Figure 2) using the pB7GWIWG2::FaNPR32-RNAi silencing construct, highly predicted to produce siRNAs targeting essentially *FaNPR3.2* alleles. Transient reduction in either all *FaNPR3.1*, *FaNPR3.2*, and *FaNPR3.3* transcripts or mainly *FaNPR3.2* transcripts, led to a clearly noticeable decrease in disease symptoms and significantly reduced internal strawberry fruit tissue damage after *C. acutatum* inoculation (Figure 3C and Figure 4C, left). On the contrary, transient overexpression of either *FaNPR3.1* or *FaNPR3.2* genes did not significantly affect fruit susceptibility to *C. acutatum* (Figure 3C and Figure 4C, right). According to previously published results and consequently with the agroinfiltration methodology used, no clear difference in the external surface damage was detected between silenced (or overexpressed) and control fruit samples [48].

Interestingly, the reduction in the internal fruit tissue damage was more relevant after the silencing of all members of the *FaNPR3* gene family than only *FaNPR3.2*, evidencing that the *FaNPR3* paralogs negatively modulate strawberry fruit defense against *C. acutatum*. Supporting this idea, it has also been recently shown that ectopic expression of the *FvNPRL-1* (the homolog of *FaNPR3.1* in *F. vesca*) in wild-type Arabidopsis led to plants suppressing resistance to *Pst*, suggesting that FvNPRL-1 could probably function as a negative regulator of the SA-mediated defense in this heterologous system [72]. All in all, these results are consistent with a role of the strawberry *FaNPR3* gene family, similar to that of their Arabidopsis orthologs, AtNPR3/4, as negative regulators of plant defense [59].

### 3.2. FaNPR3.2 Negatively Modulates Resistance in Arabidopsis

Analysis of gene knockout mutants constitutes a prioritized and direct approach in revealing and clarifying gene function. However, so far, there are no known strawberry varieties with *FaNPR3*-knockout genes. Thus, to understand the roles of FaNPR3 members in the defense response, a heterologous expression system approach was used. We have previously used this procedure to successfully carry out functional studies of strawberry defense-related genes in Arabidopsis [44]. Accordingly, Arabidopsis *npr3npr4* double mutant plants, lacking FaNPR3.2 orthologues, that display enhanced resistance against virulent *Pst* [55], were complemented with its strawberry counterpart. Convincingly, the enhanced disease resistance of Arabidopsis double mutant *npr3npr4* to virulent *Pst* is fully complemented by overexpression of *FaNPR3.2*; this is because the resistance phenotype in the Arabidopsis *npr3npr4*::FaNPR3.2 plants, in terms of disease symptoms development and bacterial growth, resembles that of the wild-type plants (Figure 6A,B,D). Similarly, ectopic expression of this strawberry gene *FaNPR3.2* in the WT background resulted in plants (WT::FaNPR3.2) that exhibit no appreciable differences in the resistance phenotype compared with WT plants. These results strongly support the aforementioned role of the strawberry FaNPR3.2 protein, similar to that proposed in Arabidopsis for NPR3/NPR4 in negatively regulating resistance [59]. This is not surprising given the high amino acid sequence similarity and the existence of well-conserved motifs between the FaNPR3.2 protein and its Arabidopsis orthologs [49,72,73].

Curiously, overexpression of FaNPR3.2 in the Arabidopsis WT and *npr3npr4* background results in increased cell death in response to *Pst* infection compared to untransformed WT and double mutant *npr3npr4* plants, especially in the WT background where they coexist with Arabidopsis proteins AtNPR3 and AtNPR4 (Figure 6C). However, it does not appear to have an appreciable effect on restricting bacterial growth (Figure 6D). In this sense, our results agree with an additional role for FaNPR3.2 as positive regulator of cell death in Arabidopsis throughout the defense response to *Pst*, as it has been reported for AtNPR3/4. Consequently, the double mutant *npr3npr4* suppresses cell death in response to avirulent pathogen infections [74].

### 3.3. Silencing of FaNPR3 Members in Strawberry Fruit Downregulates FaWRKY19 and FaWRKY24 Gene Expression

In an attempt to expand our understanding of potential molecular players within the strawberry regulatory defense network downstream of FaNPR3, we monitored the molecular signature of several strawberry SA- and JA-responsive genes that are known to be up-regulated after *C. acutatum* infection [42,45]. Only the transcript accumulation of the *WRKY* genes *FaWRKY19* and *FaWRKY24* [50] was found to be significantly reduced in the fruits after agroinfiltration with both *FaNPR3.1*- and *FaNPR3.2*-silencing constructs (Figure 4), and no relevant change was detected in the expression level for the remaining SA- and JA-responsive strawberry genes analyzed.

Unexpectedly, and in contrast to the transcriptional co-repressor role described for its Arabidopsis *NPR3/4* orthologs [59], our results reveal that, in strawberry fruit, members of the FaNPR3 clade positively regulate *FaWRKY19* and *FaWRKY24* genes. Very intriguingly, the concomitant decrease in transcript accumulation of both genes following the silencing of *FaNPR3* members correlates with the decrease in tissue damage observed in fruit after *C. acutatum* inoculation. This result is consistent with our previous report, stating that the silencing of the *FaWRKY24* gene (previously reported as *FaWRKY1*) in strawberry fruit increases the resistance of this tissue to *C. acutatum* infection [44]. In fact, *WRKY* genes are well known to modulate defense responses either positively or negatively [75]; in *F. ananassa*, down-regulation of other members of the *WRKY* family such as *FaWRKY29* or *FaWRKY64* has also been correlated with enhanced resistance against pathogens [52]. Interestingly, the strawberry FaWRKY19 protein shows a high degree of amino acid similarity with Arabidopsis AtWRKY25/33/26 proteins [50] and WRKY25/33 orthologs have also been correlated with increased plant resistance. The AtWRKY33 protein has been described as a key component of the defense-related pathway in plants, which exhibits a complex and contradictory functional role. Indeed, silencing of *AtWRKY33* gene led to plants with increased susceptibility to necrotrophic fungal pathogens [76]; but, also, the ectopic expression of this gene caused enhanced susceptibility of plants to the bacterial pathogen *Pst* [77], suggesting that this AtWRKY33 protein can act either positively or negatively on plant defense, depending on the pathogen lifestyle [78]. Consistent with our results in strawberry, the down-regulation of *WRKY33* orthologs in rice led to enhanced resistance to *Xanthomonas oryzae* [79]. Similarly, *AtWRKY25*-overexpressing plants display increased bacterial growth and enhanced disease symptoms, while silencing of *AtWRKY25* reduced disease symptoms after *Pst* infection [80]. These reports highlight the idea that different plant species can assemble slightly different molecular mechanisms in response to pathogens, and that components of the plant defense network can exhibit a versatile way of acting depending on the lifestyle of the attacker encountered [78,81].

In strawberry, it remains to be further studied whether FaNPR3 members control downstream defense-response components differently to their orthologs in Arabidopsis and/or whether this protein acts as a molecular switch when the type of pathogen differs. Curiously, based on the structural differences between strawberry NPR-like proteins and their orthologs in Arabidopsis, it appears that functional divergence may occur in strawberry NPRs orthologs [49].

### 3.4. Resistance to Pseudomonas syringae in Arabidopsis Plants Overexpressing FaNPR3.2 Is Uncoupled from PRs Gene Expression

Our results, showing the recovery of *npr3npr4* mutant plants resistance phenotype to wild-type levels, in terms of disease development, cell death, and bacterial growth, by ectopic expression of *FaNPR3.2* (Figure 6), strongly support that FaNPR3.2 and AtNPR3/AtNPR4 proteins have similar functions in plant defense. To further extend our knowledge about the role of FaNPR3.2, we monitored the expression pattern of the classical *PR* markers (a hallmark of the SA signaling pathway) [55] in Arabidopsis plants overexpressing *FaNPR3.2* and compared them with those in WT and *npr3npr4* double mutant plants.

Paradoxically, our data reflect the uncoupling between *PR*s transcript accumulation and the resistance phenotype. Indeed, overexpression of *FaNPR3.2* in both *npr3npr4* and WT genetic backgrounds results in higher constitutive expression levels for this set of *PR* genes, that are further induced upon *Pst* infection (Figure 7). Interestingly, the uncoupling between SA-dependent markers expression profiles and the resistance phenotype has been reported previously by our group when complementing the Arabidopsis mutant *wrky75* by overexpressing strawberry FaWRKY1 [44]. Furthermore, the uncoupling between SA markers and plant resistance has also been previously reported by Zeier et al., 2004 [82], where it was shown that systemic acquire resistance can be executed independently from *PR*s markers under specific external conditions.

At this point, explaining the uncoupling mechanism driven by *FaNPR3.2* would be speculative. However, our results show that the *AtNPR1* transcript level in the absence of infection is similar in WT, *npr3npr4* mutant, and overexpressing FaNPR3.2 plants (Figure 8A, upper panel); also, the level of *AtNPR1* induction in *Pst*-challenged plants does not differ significantly among all the Arabidopsis lines (Figure 8B, upper panel). In contrast, as expected, *AtNPR3* and *AtNPR4* transcripts are absent in *npr3npr4*::FaNPR3.2 lines (Figure 8A, middle and lower panels); but interestingly, the transcript basal level of these two Arabidopsis genes is reduced in WT::FaNPR3.2 compared to that of wild-type plants (Figure 8B, middle and lower panels). This last finding is very revealing, since the *FaNPR3.2* overexpression in Arabidopsis correlates with a decrease in the levels of the two well-known negative coregulators of *PR* expression [59], which could explain the high constitutive level of *PR*s observed in these plants (Figure 7). Also, AtNPR3/4 have been described as Cullin 3 RING ubiquitin ligases adaptors mediating NPR1 degradation [60]. Thus, the reduced basal level of NPR3/4 in the WT::FaNPR32 overexpressing background is consistent with the reduced degradation of the positive defense regulator AtNPR1, which also could contribute to the higher *PR*s basal level detected in those plants.

Interestingly, in response to *Pst* infection, *AtNPR3* and *AtNPR4* transcript levels are significantly induced and restored to wild-type levels in *FaNPR3.2*-overexpressing plants (Figure 8B). This result is evidence that the repression that FaNPR3.2 exerts on *AtNPR3* and *AtNPR4* genes is abolished in response to the pathogen, which suggests that FaNPR3.2 may be sensitive to redox changes and/or to variations in SA levels related to the plant response to infection, like its Arabidopsis orthologs. In addition, it is known that, under those conditions, the master regulator NPR1 is not the only substrate for NPR3/4- mediated degradation [53]. NPR3/4 proteins can also bind and act as adaptors to other key regulatory proteins that mediate immune responses, such as the JA transcriptional repressor JAZ1, targeting them towards the Cullin 3 RING ubiquitin ligase-mediated degradation pathway [60]. In fact, the AtNPR3/4-mediated SA-dependent degradation of JAZs makes it possible to tailor an efficient specific defense response against biotrophic pathogens without compromising resistance to necrotrophic pathogens, due to the activation of the JA-signaling pathway [61]. Our data on the role of FaNPR3.2 in Arabidopsis are reminiscent of that of AtNPR3/4 in the crosstalk between the SA and JA signaling pathways to prioritize one over the other and thus adjust the response against necrotrophic or biotrophic pathogens as necessary [83]. Although more research is needed to clarify this aspect, the results presented here support a dual role for FaNPR3.2 in Arabidopsis as negative regulators of AtNPR3 and *AtNPR4* genes and the defense response, as well as being modulators of the extent of the immune response after infection.

On the other hand, our results are not in concordance with those described by Shu et al. [72], reporting that the ectopic expression of *FvNPRL-1* in Arabidopsis wild-type seedlings suppressed SA-mediated *PR1* expression. To further investigate this discrepancy, we grew Arabidopsis WT, *npr3npr4* double mutant, and their corresponding FaNPR3.2-overexpressing lines on MS plates with or without SA and monitored the *PR*s expression pattern in those seedlings. As shown in Supplementary Materials Figure S1, all seedling lines analyzed displayed similar *PR1*, *PR2*, and *PR5* expression patterns in response to SA treatment profiles to that observed previously in plants upon *Pst* infection. These results strengthen the positive correlation observed in plants between *FaNPR3.2* overexpression and the higher constitutive basal and *Pst*-induced levels of *PR*s, and they differ notably from those reported by Shu et al. 2018. Although intriguing, this inconsistency can be attributed to the specific NPR3 alleles overexpressed, *FvNPR3.1*, from *F. vesca*, vs. *FaNPR3.2*, from *F. ananassa*. Indeed, the putative hinge region (LENRV) in the SA-binding core is conserved in the FvNPR3.1 protein and in all members in the strawberry FaNPR3.1 and FaNPR3.3 clade but not in FaNPR3.2 (FENRV) (Supplementary Materials Figure S3) [49,72]. Also, this region is conserved in AtNPR1 (LENRV) but differs in AtNPR3 and AtNPR4 (LEKRV). It has been described that punctual amino acid differences in this region conduct to slight differences in the SA-binding capacity of AtNPR4 and its interactions with AtNPR1 [73,84].

The plant immune system is a complex mechanism comprising a highly connected molecular network whose components can exhibit diverse functions to refine a successful plant defense against different pathogens. Unraveling the interaction of FaNPR3 members and downstream components of the different defense-related pathways in strawberry represents a challenge and an exciting task that will help to engineer broad-spectrum disease resistance in this important crop.

## 4. Materials and Methods

### 4.1. Biological Material, Growing Conditions, and Pathogen and Elicitor Treatments

Strawberry fruit (*Fragaria* × *ananassa* cv. Primoris) was harvested and grown under field conditions at the Experimental Farm “El Cebollar”, IFAPA (Huelva, Spain). Fruits were collected with pedicel (about ten centimeters long) at an early red stage with a degree of pigmentation of about 25% as described in [85], sterilized with commercial bleach (1:60 *v*/*v*), and kept individually with the pedicel immersed in sterile MS medium (0.25% Murashige Skoog) supplemented with 0.4% (*w*/*v*) sucrose, throughout the whole assay period (7 days). The MS medium was replaced every two days to minimize the effect of water stress. Fruits were kept at 25 °C, with a photoperiod of 16 h light/8 h dark.

*Colletotrichum acutatum* strain CECT 20240 was used for the fruit inoculation experiments. *C. acutatum* was grown at 20 °C with 16/8 photoperiod, on strawberry agar (500 g/L liquefied strawberry berries and 1.5% bacteriological agar) to improve the infectivity of the pathogen. For pathogen inoculations, the starting conidia suspensions (10^5^ conidia/mL) were prepared by diluting a stock of conidia, previously obtained by scraping the surface of mycelia grown for 4 weeks, in sterile distilled water (0.03% Tween-80), filtering it through glass wool, and counting cells in a Neubauer Chamber.

The *Arabidopsis thaliana* plants used in this study belong to the Columbia (Col-0) ecotype. Wild-type control plants (WT) (Nottingham Arabidopsis Stock Centre, N1093), *npr3npr4* double-knockout mutant plants (provided by X. Dong, Duke University, Durham, NC, USA), and the transgenic lines generated in this work were germinated and grown under controlled conditions (22 °C, 50% humidity, 9/15 h photoperiod at a light intensity of 125 mol m^−2^ s^−1^), as previously described [44].

The Arabidopsis infection experiments were performed with 3–4-week-old plants using *Pseudomonas syringae* pv. tomato virulent strain DC3000 (*Pst*) provided by Dr. Antonio Molina (CBGP-UPM, Madrid, Spain).

For the transient ectopic expression and silencing experiments in the strawberry fruits, *Agrobacterium tumefaciens* strain AGL0 was used. For the stable transformation of Arabidopsis plants, the *A. tumefaciens* strain GV3101 was used.

For the salicylic acid (SA) treatment, Arabidopsis seeds were sterilized for 15 min (70% ethanol, 0.05% Triton), and then grown on solid Murashige–Skoog (MS) supplemented with 1% sucrose and 0.4% phytagel, under the controlled conditions mentioned above. Twelve-day-old Arabidopsis seedlings were transferred to equivalent medium containing 200 μM SA or a MS medium without SA (control) and incubated for 24 h, as described by Shu et al., 2018 [72].

### 4.2. Plasmid Construction for Silencing and Overexpressing FaNPR3 Genes

For silencing, binary plasmids pFRN (courtesy of Dr. Marten Denekamp, Department of Molecular Cell Biology, University of Utrecht (Utrecht, The Netherlands) and pB7GWIWG2.0 vector (VIGS-Plant Systems Biology, Ghent, Belgium) were used. Standard Invitrogen protocols were used for the cloning steps using gateway technology. For the silencing of all members of *FaNPR3.1*, -*3.2*, and -*3.3* the 407 bp DNA candidate sequence described in Súnico et al., 2022 [40], was cloned into pCR8/GW/TOPO (Invitrogen, Carlsbad, CA, USA)) and subsequently transferred to pFRN destination vector to obtain the RNAi silencing construct pFRN::FaNPR3*all*.RNAi. For the silencing of *FaNPR3.2* members, a 535 bp DNA candidate sequence (Supplementary Materials Figure S2) from the 5′UTR region of the *FvNPR3.2* gene was amplified by PCR and cloned into pDONR221 (InvitrogenTM, Carlsbad, CA, USA), and subsequently transferred to pB7GWIWG2.0 destination vector to obtain the RNAi silencing construct pB7GWIWG2::FaNPR32.RNAi. Candidate sequences were selected and amplified from *Fragaria* × *ananassa* genome using www.invivogen.com/sirnawizard/design.php (accessed on 15 September 2021) and http://sirna.wi.mit.edu (accessed on 15 September 2021), as previously described [40]. The presence of the sense and antisense orientation of the candidate DNA fragments spaced by the CHS intron was confirmed by sequencing. For the ectopic expression of FaNPR3.1, a 1.764 kb cDNA fragment (Supplementary Materials Figure S3), carrying the complete FaNPR3.1 ORF sequence, was cloned into pENTR/D/TOPO (InvitrogenTM) and then transferred to pK7WG2.0 destination vector [86]. For the ectopic expression of FaNPR3.2, a 1.785 kb cDNA fragment (Supplementary Materials Figure S4), carrying a complete FaNPR3.2 ORF sequence, was cloned into pDONR221 (InvitrogenTM) and then transferred to a pB7WG2 destination vector [86]. Both final constructs, pK7WG2::FaNPR31.OE and pB7WG2::FaNPR32.OE, respectively, carry the inserted genes under the control of the CaMV35S promoter. All cDNA fragments were obtained from *Fragaria ananassa* using total RNA as template. Primer information is provided in Supplementary Materials Table S1. All DNA inserts were sequenced prior to further manipulations.

All constructs were introduced into the *A. tumefaciens* strains indicated above. For the transient ectopic expression and silencing experiments in strawberry fruit, the *A. tumefaciens* strain AGL0, carrying the pK7WG2::FaNPR31.OE, pB7WG2::FaNPR32.OE, pFRN::FaNPR31*all*.RNAi, and pB7GWIWG2::FaNPR32.RNAi constructs, respectively, and their corresponding empty vectors, was used. For the stable transformation of Arabidopsis plants, the *A. tumefaciens* strain GV3101 carrying the pB7WG2::FaNPR32.OE construct was used [87].

### 4.3. Agroinfiltration of Strawberry Fruit and Experimental Design

To analyze the effect of the silencing of *FaNPR3* genes on the defense response to *C. acutatum* inoculations, we confronted a query silencing situation to its non-silenced control within the same fruit, thus avoiding the existing variability among the strawberry fruits used in the assay. Thus, one half of the fruit was infiltrated with *A. tumefaciens* (agroinfiltration) carrying the silencing/overexpression construct and the opposite half was infiltrated with *A. tumefaciens*, carrying the corresponding empty vector, as a control. Then, we compared the defense response to *C. acutatum* inoculations between halves of the same fruit. The complete and detailed protocol of this agroinfiltration procedure has been previously described in Higuera et al., 2019 [48]. Briefly, for each condition and gene, a set of 144 strawberry fruits were agroinfiltrated; two days later, 120 of them were inoculated with *C. acutatum* in both halves, leaving the remaining 24 fruits to analyze the silencing of each gene under no infection conditions. Of the 120 inoculated fruits, 24 were used for gene expression studies and the 96 remaining fruits were used for tissue damage evaluation and statistical purposes. In accordance with Higuera et al., 2019 [48], the whole assay was repeated twice during strawberry fruiting season for two years. Samples were collected from each of the two halves of three fruits at different times for 7 days after agroinfiltration (7 dai), were quickly frozen with liquid nitrogen, and kept at −80 °C, to be used later for the gene expression analysis by RT-qPCR. Likewise, every year, fruit tissue damage was evaluated at 6 dai, which corresponds to 4 days post-inoculation with *C. acutatum* (4 dpi), both in 48 infected fruits, previously agroinfiltrated with the silencing construct in one half of the fruit and the empty vector in the opposite half, and in 48 infected fruits where both halves were agroinfiltrated with the empty vector, as a control for statistical purposes [48]. The effect of ectopic expression of *FaNPR3.1* and *FaNPR3.2* genes in the response of strawberry fruits to *C. acutatum* inoculation was evaluated following an identical protocol and experimental design but monitoring tissue damage evaluation after fruit agroinfiltration with *A. tumefaciens* strains bearing the constructs pK7WG2::FaNPR31.OE and pB7WG2::FaNPR32.OE, respectively, and their corresponding empty vectors.

### 4.4. Stable Transformation of Arabidopsis Plants

Arabidopsis wild-type and *npr3npr4* mutant plants were transformed with the *A. tumefaciens* GV301 strain containing both the construct pB7WG2::FaNPR32.OE and its corresponding empty vector using the floral dip procedure [88]. Seeds from the transformed plants (T1) were harvested, sown, and selected based on their resistance to BASTA herbicide (ammonium glufosinate, Bayer CropScience, Dublin, Ireland) by spraying 2-week-old plants 3 times every 48 h with a solution containing 1.2% BASTA, 0.05% L-77 silwet (Phytotechnology Laboratory, Lenexa, KS, USA). The resulting progeny (T2), segregated in a 3:1 ratio (BASTA-resistant/-susceptible) were selected to carry a single insertion; homozygous lines (T3) were obtained from at least 10 individuals for each construct and confirmed for homozygosis in the offspring for the expression of the transgene by RT-PCR using the specific primers (Supplementary Materials Table S1) and selected for further analyses.

### 4.5. Arabidopsis Infection Assay with Pseudomonas syringae

*Pst* growth, plant inoculation, and in planta bacterial growth analyses were performed as described previously with 10^6^ CFU mL^−1^ in 10 mM MgCl_2_ and were pressure-infiltrated into the leaves [89]. For monitoring cell death, 12 leaves per genotype and treatment were collected 24 h after inoculation in the control and the *Pst*-infected leaves and stained using Tripan Blue, as previously described [90]. All the experiments were repeated at least three times using ten plants per experiment and treatment.

### 4.6. RNA Extraction and Real-Time qPCR

Total RNA from frozen independent halves of agroinfiltrated strawberry fruits was extracted with the Maxwell ^®^ 16 LEV Plant RNA kit (Promega, Madison, WI, USA), according to the instructions provided by the manufacturer. Arabidopsis total RNA was extracted using 50 mg leaf sample from 12-day-old seedlings (4 biological replicates each consisting of seedlings grown in the same plate) or 2–3-week-old soil-grown plants (4 biological replicates each consisting of leaves from 3 plants), using the Invitrap Spin Plant RNA MiniKit (Invitek Molecular, Berlin, Germany); contaminating genomic DNA was removed by DNase I (Invitrogen) treatment. Purified RNA was quantified spectrophotometrically on NanoDrop 1000 (Thermo Scientific, Waltham, MA, USA) and RNA integrity (RIN) was verified using the Agilent 2100 Bioanalyser (Agilent Technologies, Waldbronn, Germany). Reverse transcription (RT) was carried out using 250 ng and 2 µg of purified total RNA as template from strawberry and Arabidopsis samples, respectively, with an RIN value ≥ 8 following manufacturer instructions [iScript cDNA synthesis kit (Bio-Rad, Hercules, CA, USA)]; then, 50 ng cDNA were used for RT-qPCRs using SsoAdvancedTM SYBR ^®^ Green Supermix, MyIQ v1.004 and iCycler v3.1 real-time PCR systems (Bio-Rad) and specific primers for each of the genes tested with similar PCR efficiencies. Three technical replicates in the same run and three–four biological replicates in different runs were performed, as described in Encinas-Villarejo et al. 2009 [44]. All primers for qRT-PCR used in this study are listed in Supplementary Materials Table S1.

Relative expression values were determined by the 2^−ΔΔCt^ method [91], using as internal standards the housekeeping genes actine 1 (*FaACT1*) and elongation factor 1α (*FaEF1a*) for strawberry [92], and *ACTIN2* for Arabidopsis [44]. One-way ANOVA followed by HSD Tukey’s testing was computed using R functions “lm”, “anova”, and “TukeyHSD”.

### 4.7. Assessment and Statistical Analysis after Pathogen Infection

For both the silencing and the ectopic expression experiments in strawberry, a phenotypic evaluation was performed to determine fruit tissue damage after 4 days post-inoculation (4 dpi) with *C. acutatum*, corresponding to 6 days after agroinfiltration (6 dai). For statistical purposes, fruits with both halves agroinfiltrated with the empty vector were used as control. Per year, a sample of 48 half fruits for silencing/overexpression, and their corresponding 48 control half fruits, were evaluated for internal damage, according to a scale of 1 to 5 (1, 0% damage, asymptomatic tissues; 2, up to 10% damage, weak injury; 3, between 10 and 25% damage, moderate injury; 4, between 25 and 50% damage, severe injury; 5, more than 50% damage, severely affected fruit), as described in Higuera et al. (2019) [48]. Therefore, the internal damage ratio was calculated by dividing the tissue damage value of the fruit half where the gene was silenced/overexpressed by the tissue damage value corresponding to the opposite half of the same fruit, agroinfiltrated with the empty vector. Means and SE were calculated by Fisher’s LSD (α = 0.05) and Statistix software (v9.0). A ratio value of 1 indicates no difference between the two halves of the same fruit.

The statistical analysis of the bacterial growth data was performed according to Encinas-Villarejo et al. 2009 [44].

## 5. Conclusions

We have shown that *FaNPR3* members in strawberry act as negative regulators of resistance to *C. acutatum* and as positive regulators of the *FaWRKY19* and *FaWRKY24* defense genes. Furthermore, our results in Arabidopsis agree with this role of *FaNPR3.2* as a negative regulator of defense responses; in addition, they evidence a positive effect of this strawberry gene on the induction of the classic *PR* resistant marker genes in this heterologous system. The novel results presented in this work highlight strawberry *FaNPR3* members as promising candidates for new environmentally friendly breeding technology strategies to accelerate strawberry resistance improvement, while minimizing fitness costs.

## Data Availability

The original contributions presented in the study are included in the article/Supplementary Materials, further inquiries can be directed to the corresponding authors.

## Supplementary Materials

The following supporting information can be downloaded at: https://www.mdpi.com/article/10.3390/plants13162261/s1, Figure S1: Expression of the defense-related genes *PR1*, *PR2*, and *PR5* in *Arabidopsis* SA-treated seedlings. Relative expression level was monitored by qRT-PCR in control and SA-treated seedlings 1 day post treatment (200 μM) in WT, WT::FaNPR32, double mutant *npr3npr4*, and *npr3npr4*::FaNPR32 (black, grey, white and striped bars, respectively). Expression levels were normalized with respect to the internal control *ACTIN2* and displayed relative to the expression in mock-treated samples that were given a value of 1. Bars refer to mean ± standard error. Note the different scales in the relative-expression-level axis. Statistical significance was determined by one-way ANOVA. Letters indicate significant differences (*p* < 0.05) in HSD Tukey’s post hoc test. Figure S2: The 533bp *FvNPR3.2* cDNA fragment for RNAi silencing. Position of the forward attB1-533RNAi and attB2-533RNAi reverse primers, are labelled in red-dark green and redlight green, respectively. Primer sequences are listed in Supplementary Table S1. Figure S3: *FaNPR3.1* nucleotide coding sequence (A) and its corresponding amino acid sequence (B). (A) The start codon (ATG) and the stop codon (TGA) are in italics, underlaid and colored in blue; primers for cDNA cloning are red-dark green (forward) and red-light green (reverse); additional sequences for cloning purpose are in red; the position of primers for gene expression analyses are underlaid. (B) amino acid residues corresponding to NPR conserved domains are labelled in different colors as follow, BTB superfamily domain, in green; DUF3420 domain, in pink; superfamily “NPR1-like-C”, in red; ANK superfamily domain is boxed in blue. The putative hinge region (LENRV) in the SA-binding core is highlighted in yellow box with larger, bolder font size. Figure S4: *FaNPR3.2* nucleotide coding sequence (A) and its corresponding amino acid sequence (B). (A) The start codon (ATG) and the stop codon (TGA) are in italics, underlaid and colored in blue; primers for cDNA cloning are red-dark green (forward) and red-light green (reverse); attB sequences are in red; the position of primers for gene expression analyses are underlaid. (B) amino acid residues corresponding to NPR conserved domains are labelled in different colors as follow, BTB superfamily domain, in green; DUF3420 domain, in pink; superfamily “NPR1-like-C”, in red; ANK superfamily domain is boxed in blue. The putative hinge region (FENRV) in the SA- binding core is highlighted in yellow box with larger, bolder font size. Table S1: List of primers used in this work.
