# Supplementary material for: FaNPR3 Members of the NPR1-like Gene Family Negatively Modulate Strawberry Fruit Resistance against Colletotrichum acutatum
Source: Plants (Basel). 2024 Aug 14;13(16):2261. doi: 10.3390/plants13162261 (PMC11360474; doi:10.3390/plants13162261)

Supplementary Figure S1. Expression of the defense-related genes *PR1*, *PR2*, and *PR5* in *Arabidopsis* SA treated seedlings. Relative expression level was monitored by qRT-PCR in control and SA treated seedlings 1 day post treatment (200µM) in WT, WT::FaNPR32, double mutant *npr3npr4*, and *npr3npr4*::FaNPR32 (black, grey, white and striped bars, respectively). Expression levels were normalized with respect to the internal control *ACTIN2* and displayed relative to the expression in mock-treated samples that were given a value of 1. Bars, refer to mean ± standard error. Note the different scales in the relative expression level axis. Statistical significance was determined by one-way ANOVA. Letters indicate significant differences ($p < 0.05$) in HSD Tukey's post-hoc test.

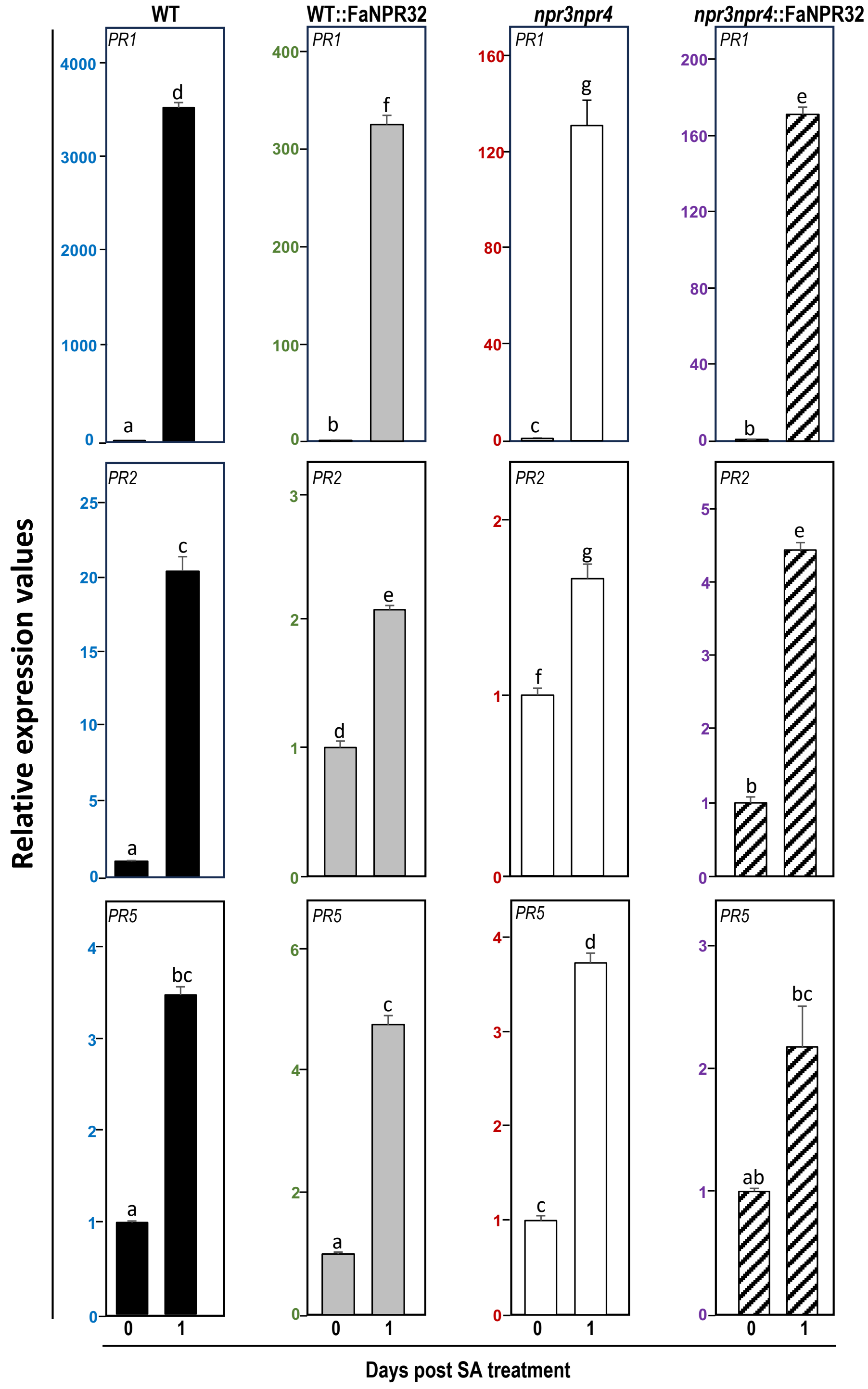

Supplement: Supplementary file 1 [file plants-13-02261-s001.zip › plants-3135816-supplementary/SUPPLEMENTARY FIGURES AND TABLES/SUPPLEMENTARY FIGURE S1.pdf]
