# Supplementary material for: FaNPR3 Members of the NPR1-like Gene Family Negatively Modulate Strawberry Fruit Resistance against Colletotrichum acutatum
Source: Plants (Basel). 2024 Aug 14;13(16):2261. doi: 10.3390/plants13162261 (PMC11360474; doi:10.3390/plants13162261)
Supplement: Supplementary file 1 [file plants-13-02261-s001.zip › plants-3135816-supplementary/SUPPLEMENTARY FIGURES AND TABLES/SUPPLEMENTARY FIGURE S2.pdf]

Supplementary Figure S2. The 533bp *FvNPR3.2* cDNA fragment for RNAi silencing. Position of the forward attB1-533RNAi and attB2-533RNAi reverse primers, are labelled in red-dark green and red-light green, respectively. Primer sequences are listed in Supplementary Table S1.

GGGGACAAGTTTGTACAAAAAAGCAGGCTTCCATCTTTGAGGAGAAGTGCGGCTTTTGCTATATTCG
GTCAATACCATTGAGATCTTCTTGGAAGCTGTCAAGCTGCTGCTTCAACTTGTTCAAGTCCTCATTGAATT
TCATAGCATCTTTAAGAAGGAGAATAGTCCTTGGTTGATCAGAAGGCTCAGCCTCATCTTCTTCATCATCT
CAAGCTGTGTTGCTGTTCACTAAGCTTAAAAGTCAAAAGGGTGCGTCTTAATCTGTACGCAGTCTTTCTC
CCTTTGTGCTTTTTGTCATTAATTTTTAAGGGTTGAAGGGTGGTTTGTGGTTTCTGAGTTTGAGTAGTGCAA
GGTTTGGTTTTGATGAAGATCCAAGTCATATGTGTATTGGGAAGTTCAGGATTTACTACCTCAAGTTGTGT
ACCTAGCTCTACTCAGAAATTGTTCTTGGGATTTTGCTTTTGGATGAATTCTCAAACTGGGTCAGCTTAGT
ATGCTTTTATCTGTTACATTATGCAGGCTTTGCATCAGAAATGATGATATGGTCGTCTTATTAAGGGGGCT
ACCCAGCTTTCTTGTACAAAGTGGTCCC
