# Supplementary material for: FaNPR3 Members of the NPR1-like Gene Family Negatively Modulate Strawberry Fruit Resistance against Colletotrichum acutatum
Source: Plants (Basel). 2024 Aug 14;13(16):2261. doi: 10.3390/plants13162261 (PMC11360474; doi:10.3390/plants13162261)
Supplement: Supplementary file 1 [file plants-13-02261-s001.zip › plants-3135816-supplementary/SUPPLEMENTARY FIGURES AND TABLES/SUPPLEMENTARY FIGURE S3.pdf]

Supplementary Figure S3. *FaNPR3.1* nucleotide coding sequence (A) and its corresponding amino acid sequence (B). (A) The start codon (ATG) and the stop codon (TGA) are in italics, underlaid and colored in blue; primers for cDNA cloning are red-dark green (forward) and red-light green (reverse); additional sequences for cloning purpose are in red; the position of primers for gene expression analyses are underlaid. (B) amino acid residues corresponding to NPR conserved domains are labelled in different colors as follow, BTB superfamily domain, in green; DUF3420 domain, in pink; superfamily "NPR1-like-C", in red; ANK superfamily domain is boxed in blue. The putative hinge region (LENRV) in the SA-binding core is highlighted in yellow box with larger, bolder font size.

(A)

CACC*ATG***GCGAATTCAGGTGAGCC**ATCGTCTTCTTTGAGCTTCACTTCATCTTCCCATCTATCAA
ATGGTTCGATAAGCCACAACTTATCTTCCTCTGAAGCCTTGCCTAGTCTTGAAGTCATCAGTTTGA
CCAAGCTTAGCTCTAGTTTGGAGCAGCTCTTGCTTGATTCTGGCTGTGATTATAGTGATGCCGAA
ATCATTGtTGAGGGTGATCCTGTTGGTGTTCACCGATGTATATTGGCTTCTAGGAGCAAGTTTTTT
GTGAACTATTTAAGAAAGAAAAGGGGTATTCTGAGAAGGAGGGCAAACTGGGGAAACCAAAGTA
TAAAATGAAGGATTTGCTACCTTATGGGGACGTTGGATATGAAGCATTTTTGGTTTACTTGAACTAC
GTGTATACCGGAAAGCTGAAGCCTTCTCCCATTGAGGTGTCAACTTGTGTGCACAATGTATGTGC
CCATGACTCATGTAGACCTGCAATCAATTTTGCTGTGGAATTGATGTACGCCTCTTCCATTTTCCA
AATGCCGGATTTGGTTTCAGTATTCCAGCGACGCCTTCTTAATTTTGTTGGGAAAGCTATGGCAG
AAGATGTCATCCCAATCCTTGTGGTTGGTTTCCACTGTCATTTGAGCGAGCTTGTCACTCATTCCA
TAGAGAGAGTGGCACGATCAGATCTTGATAGCATCTGTATTGACAAGGAACTTCCTTATGAGGTT
GCAGAGAAAATTAAACTGCTTCGTCGTGATTCTCAACATGCTAGTGAACTGAGCACTCCACCTGT
GGACCCCTTGCGTGAAAAAAGAATTAGGAGCATACATAAGGCATTAGACTCGGATGATGTTGAA
CTTGTGAAACTTCTTTTATCTGAGTCGAATATAACCTTAGATGAAGCCCATGCTCTCCACTATGCTG
CAGCCTACTGTGATCCTAAGGTTGTTACTGAAGTTCTTGGTCTGGGTATGGCTGATGTTAACCTTC
GTAATCCTCGGGGTTATACCGTGCTTCATATTGCTGTGATGCGCAGAGAGCCATCAATAATAGTAT
TGCTTCTTAGTAAAGGAGCTCGTGCATCAGAGCTGACATCAGATGGTCAAAGTGCAGTTAGTATT
TGCCGGCGGtTGACAAGGGCAAAGGACTATCATTCAAAAACAGTGCAGGGGCAAGAAACTAA
CAAGGACCGAATTTGCATTGATGTTCTAGAGAGAGAAATGCGGAAGAATCCAATGGCAGGAGAT
GCATCTGTGTCTTCTCAAACAATTGCTGATGATCTGCACATGAAATTGCTGTACCTGGAGAACAG
AGTGGCTTTTGCTCGTTTGTTTTTCCCTAGTGAAGCAAAGCTAGCCATGGTCGTTGCTCAATCCG
ACACAACATCTGAGTTTGCTGGAATCTCTCCATCAAAAGGCTCAAGCGGGAATTTGATGGAGGT
TGATTTAAACGAGACCCCCACAGTGCAGAAGAAAAGACTCCATTCTAGGTTGGAATCCCTTATG
AAAACAGTCAACATGGGTAGATGCTATTTCCCTCATTGCTCAGAAGTCCTGGATAAGTTTATGGAG
GATGACCTGCCTGATTTGTTCTTTCTTGAGACGGGTACCCCTGACGAGCAGAAAATAAAGAGGA
TGCGTTTCATGGAGCTCAAGGATGAAGTCCAGAAGGCATTTAGCAAGGACAAAGCGGAGCTCA
ACCTCTCTGGGTTGACTTCTTCATCATCCTCATCATCTCCAAAAAAAGTTGGTGTAAATCACAAGA
CTAGAAAA*TAG*GGTG

(B)

MANSGEPSSSLSFTSSSHLSNGSISHNLSSSEALPSLEVISLTKLSSSLEQLLLDSGCDYSDAEIIVE
GDPVGVHRCILASRSKFFCELFKKEKGYSEKEGKLGKPKYKMKDLLPYGDVGYEAFLVYLNYVYTG
KLKPSPIEVSTCVHNVCAHDSCRPAINFAVELMYASSIFQMPDLVSVFQRRLLNFVGKAMAEDVIPI
LVVGFHCHLSELVTHSIERVARSDLDSICIDKELPYEVAEKIKLLRRDSQHASELSTPPVDPLREKRIR
SIHKALDSDDVELVKLLLSESNITLDEAHALHYAAAYCDPKVVTEVLGLGMADVNLRNPRGYTVLHI
AVMRREPSIIVLLLSKGARASELTSDGQSAVSICRRLTRAKDYHSKTVQGQETNKDRICIDVLEREM
RKNPMAGDASVSSQTIADDLHMKLLY**LENRV**AFARLFFPSEAKLAMVVAQSDTTSEFAGISPSKG
SSGNLMEVDLNETPTVQKKRLHSRLESLMKTVNMGRCYFPHCSEVLDKFMEDDLPDLFFLETGTP
DEQKIKRMRFMELKDEVQKAFSKDKAELNLSGLTSSSSSSSPKKVGVNHKTRK-
