# Supplementary material for: FaNPR3 Members of the NPR1-like Gene Family Negatively Modulate Strawberry Fruit Resistance against Colletotrichum acutatum
Source: Plants (Basel). 2024 Aug 14;13(16):2261. doi: 10.3390/plants13162261 (PMC11360474; doi:10.3390/plants13162261)
Supplement: Supplementary file 1 [file plants-13-02261-s001.zip › plants-3135816-supplementary/SUPPLEMENTARY FIGURES AND TABLES/SUPPLEMENTARY FIGURE S4.pdf]

Supplementary Figure S4. *FaNPR3.2* nucleotide coding sequence (A) and its corresponding amino acid sequence (B). (A) The start codon (ATG) and the stop codon (TGA) are in italics, underlaid and colored in blue; primers for cDNA cloning are red-dark green (forward) and red-light green (reverse); attB sequences are in red; the position of primers for gene expression analyses are underlaid. (B) amino acid residues corresponding to NPR conserved domains are labelled in different colors as follow, BTB superfamily domain, in green; DUF3420 domain, in pink; superfamily "*NPR1*-like-C", in red; ANK superfamily domain is boxed in blue. The putative hinge region (FENRV) in the SA- binding core is highlighted in yellow box with larger, bolder font size.

(A)

GGGGACAAGTTTGTACAAAAAAGCAGGCT**TAACA*ATG*GATCATATGAATGACCTTTCGTCATCTTTGA**GCTTTGC
CTCTTCTTATGTATCAAATGGATCAAGTGGTAACCATGTGTCTGCCTCGGCCAATTCTCAAAGTACGGAGCATTTGA
GTCTAAGCAGACTCAGTGATAACCTGGAGAGGCTGTTGCTTGATTCTCAATATGACTATAGTGATGCGGACATAGT
TGTTGAGGGTGTCCCAGTTGGTGTCAATCGGTGTGTATTGGCTGCCCGCAGCCAGTTTTTTCATGAGCATTTTAA
GAAGGGAAATGATGATTCGAAGAAGGAAGGCAAACCACAGTATATGATGTCTGAATTGGTTCCTTATGGTGGAG
TTGGCTGTGAAGCATTCAAGGTAGTCTTGAACTATTTGTATACTGGAAAACTTAAGCCACCACCTCCTGAAGTATC
AACTGTATCAACATGTGTTGATGATGGTTGTGCACATGATTCATGTGGACCTGCAATTAATTACGCTGTGGAATTGT
TGTATGCTGCTGCCACTTTCCAGATGAAGGAACTTGTTCAAGTTGTGCAGCGCCGTCTTACCAACTTTGTCGAGA
AAGCTCTTGTTGAAGATGTGATCCCAATTCTTGTAGCCTCTTTCCACTGCAAACAGAGTCAGCTGTTTTCACACTG
CATCCAGAGGGTAGAAAGGTCAGATCTTGACAATGTGGTTCTAGAGAAAGAGCTTCCTCATGAAGTTTTTATTAA
TATCAAATCACTCCGTCTAGAATATCAGAAAATGGCTGATTCAATTATGGTGGAAATGGAACCGGTGGAGGATAAG
AGGCTTAAAAGCATAAGAAATATCCACATGGCATTAGATACTGATGATGTTGAACTGGTCACTCGTCTTCTAGATG
AGTCTGGTTCTAGTACATTAGATGATGCTTGTGCTCTCCACTATGCTGTTGCATACTGTGATCCGAAGATTGTTAAG
GAAGTTCTTGGTCTACGTCTGGGCAATACAAACCTTCGGAATGCCCGAGGACATACTGTACTTCATGTGGCTGCA
AGGCGTAAGGACCCAGCAGTCCTAGTTCCTCTTCTGAACAGTGGAGCCTCTGCCTTAGAAATTACATCAGATGGT
CAAACTGCTGTTGCAATTTGCCGGAGGTTGACTAGACCAAAGGATTATTATGAGAACACAAAGCAGGGAGAGGT
GTCTAACAAAGATCGAATATGCATAGATCTTCTAGAGAGAGAGATGCAAAGAAATTCTATGGCTGTGAACATGTCA
AACACATCATATGTGATGGCCGATGATTTGCATGTGAGGCTGGACTATTTTGAAAATAGAGTGGCATTTGCACGGT
TGTTGTTTCCTGCTGAAGCCAAGCTTGCTATGGAAGTGGCAGATCATCCAACAACCGGGCACACTGGCCTACCAT
CAAAAGGATCTAGTGGGAACTTACGAGAGGTTGATTTGAATGAAACACCTTCTGTACGATCCAAAAGACTTCAA
GAAAAATTGCAAGCCCTTATGAAAACAGTGGAAATGGGTCGACGCTTCTTTCCTCATTGTTCAAAAGTTCTCGAT
AAGTTTCTGGATGATGAGATGGATATGGCTGATTATTTTCTTGAGAAAGGCACTCCTGAAGAGCAGAAAAACAA
GAAGATGCGCTTCTTGGAACTTAAAGATGATGTGCAGAAGGCATTTTGCAGGGATGTGGCTGCAAAACAGGGG
TCAGTCTTGACAGCCTCATCATCATCCTCATCGTCTCCAAAGGAGGGGGTTAATCGTAAGGTTAGGAAAAGGCCA
AGC*TGA*GACCCAGCTTTCTTGTACAAAGTGGTCCCC

(B)

MDHMNDLSSSLSFASSYVSNGSSGNHVSASANSQSTEHLSLSRLSDNLERLLLDSQYDYSDADIVVEGVPVGVNRCV
LAARSQFFHEHFKKGNDDSKKEGKPQYMMSELVPYGGVGCEAFKVVLNYLYTGKLKPPPPEVSTVSTCVDDGCAHD
SCGPAINYAVELLYAAATFQMKELVQVVQRRLTNFVEKALVEVIPILVASFHCKQSQLFSHCIQRVERSDLDNVVLEKEL
PHEVFINIKSLRLEYQKMADSIMVEMEPVEDKRLKSIRNIHMALDTDDVELVTRLLDESGSSTLDDACALHYAVAYCD
PKIVKEVLGLRLGNTNLRNARGHTVLHVAARRKDPAVLVPLLNSGASALEITSDGQTAVAICRRLTRPKDYYENTKQGE
VSNKDRIIDLLEREMQRNSMAVNMSNTSYVMADDLHVRLDY**FENRV**AFARLLFPAEAKLAMEVADHPTTGHTG
LPSKGSSGNLREVDLNETPSVRSKRLQEKLQALMKTVEMGRRFFPHCSKVLDKFLDDEMDMADYFLEKGTPEEQKN
KKMRFLELKDDVQKAFCRDVAAKQGSVLTASSSSSSSPKEGVNRKVRKRPS
