# Supplementary material for: FaNPR3 Members of the NPR1-like Gene Family Negatively Modulate Strawberry Fruit Resistance against Colletotrichum acutatum
Source: Plants (Basel). 2024 Aug 14;13(16):2261. doi: 10.3390/plants13162261 (PMC11360474; doi:10.3390/plants13162261)
Supplement: Supplementary file 1 [file plants-13-02261-s001.zip › plants-3135816-supplementary/SUPPLEMENTARY FIGURES AND TABLES/SUPPLEMENTARY TABLE S1.pdf]

Supplementary Table S1. List of primers used in this work.

**Fragaria ananassa Gene/Referenc**	**Primer name**
FaNPR3.1	FaN31pENT
	iQFaNPR3.1
FaNPR3.2	attB-533RNAi
	attB-FaNPR3.2
	iQ-FaNPR3.2
FaNPR3.3	iQ-FaNPR3.3
*FaPR1.1**	iQFaPR1.1
*FaPR2.1**	iQFaPR2.1
*FaPR5.2**	IQFaPR5.2
*FaWRKY24**	iQFaWRKY24
*FaWRKY19**	iQFaWRKY19
*FaACT.1**	iQFaACT-1
*FaEF1a**	iQFaEF1a

**Arabidopsis Gene ID**	**Primer name**
At3g18780	iQAtACTIN2
AT4G19660	iQAtPR1
At3g57260	iQAtPR2
At1g75040	iQAtPR5
AT1G64280	iQAtNPR1
AT5G45110	iQAtNPR3
AT4G19660	iQAt NPR4

* Information published in Amil-Ruiz et al. 2016 [45]

**Forward primer sequence (5'-3')**
CACCATGGCGAATTCAGGTGAGCC
ATAAGTTTATGGAGGATGACCTGCCT
GGGGACAAGTTTGTACAAAAAAGCAGGCTTCCATCTTTGAGGAGAAGTGCGG
GGGGACAAGTTTGTACAAAAAAGCAGGCTTAACAATGGATCATATGAATGACCTTTCGTCATCTT[illegible]
GCTGCAAAACAGGGGTCAGTCTTGA
TGGATGATCTCGGTATGGGTGATTAC
TGCTAATTCACATTATGGCG
AAGATGGACAATATGGCTACCAGA
CCAATTCCCAAATGTCCCTCATCTTGAT
ACAGCAGTAAGATTAGGGATGAAGAAGGGAG
CAAGTCAGGAGGAGGGGATGAGTA
GGGCCAGAAAGATGCTTATGTCGG
TGGATTTGAGGGTGACAACATGA

**Forward primer sequence (5'-3')**
GCCGAGATTCTTGCTCTGGA
TATGCTCGGAGCTACGCAGAA
CTCGACTCGGTTTATGCAGCA
GGCGATGGAGGATTTGAATTG
GAATCCGTCTTTGACTCGCC
TAACTGCGCTACGTCCGTCA
ATGCCCGAGGATCTCCAAAT

Reverse primer sequence (5'-3')
GTGTAAATCACAAGACTAGAAATAGGGTG
CTATTTTCTAGTCTTGTGATTTACAC
GGGACCACTTTGTACAAGAAAGCTGGGTAGCCCCCTTAATAAGACGACCATA
GGGGACCACTTTGTACAAGAAAGCTGGGTC TCAGCTTGGCCTTTTCCTAACCTTAC[illegible]
CAGCTTGGCCTTTTCCTAACCTTACGAT
TCATGTGGATGTCAAAACAGACTGGT
GTTAGAGTTGTAATTATAGTAGG
AACTCTCGCATTCTCCTTTGTAGTTA
AGTGTAAGGGCACTCGTTACGGATTTCA
GCTTCTTCACATTGCAACCCTGATGCGTG
ATCCATCATCAAGAATATCAATGTCACT
GGGCAACACGAAGCTCATTGTAGAAG
GTATACATCCTGAAGTGGTAGACGGAGG

Reverse primer sequence (5'-3')
TAACTGCGCTACGTCCGTCA
TCGCTAACCCACATGTTCACG
TTTTCCACACTCGTCCCGACT
GCGTCAAAGTTGCAGCCTGTA
GCGGTGTTGTTGGAGTCTTT
GCCGAGATTCTTGCTCTGGA
GGCTTCTGCTGGGAAGAACA

**Analysis/target**
Cloning *FaNPR3.1*
RT-qPCR/ *FaNPR3.1* expression
Silencing *FaNPR3.2* members
Cloning *FaNPR3.2*
RT-qPCR/ *FaNPR3.2* expression
RT-qPCR/ *FaNPR3.3* expression
RT-qPCR/ *FaPR1.1* expression
RT-qPCR/ *FaPR2.1* expression
RT-qPCR/ *FaPR5.2* expression
RT-qPCR/ *FaWRKY24* expression
RT-qPCR/ *FaWRKY19* expression
RT-qPCR/ internal standard
RT-qPCR/ internal standard

**Analysis/target**
RT-qPCR/ internal standard
RT-qPCR/ AtPR1 expression
RT-qPCR/ AtPR2 expression2
RT-qPCR/ AtPR5 expression
RT-qPCR/ AtNR1 expression
RT-qPCR/ AtNPR3 expression
RT-qPCR/ AtNPR4 expression
